# mRNA Vaccine Development in the Fight Against Zoonotic Viral Diseases

**DOI:** 10.3390/v17070960

**Published:** 2025-07-08

**Authors:** Brandon E. K. Tan, Seng Kong Tham, Chit Laa Poh

**Affiliations:** 1 ALPS Global Holding Berhad, The Icon, East Wing Tower Level 18-01 & Level 18-02, No.1 Jalan 1/68F, Off Jalan Tun Razak, Kuala Lumpur 50400, Malaysia; brtek96@gmail.com (B.E.K.T.); drtham@alpsmedical.com (S.K.T.); 2 Nilai University, No.1, Persiaran Universiti, Putra Nilai, Bandar Baru Nilai, Nilai 71800, Negeri Sembilan, Malaysia

**Keywords:** zoonotic diseases, viruses, mRNA vaccine, vaccine development, emerging zoonotic diseases, re-emerging zoonotic diseases

## Abstract

Zoonotic diseases are transmitted from animals to humans, and they impose a significant global burden by impacting both animal and human health. It can lead to substantial economic losses and cause millions of human deaths. The emergence and re-emergence of zoonotic diseases are heavily influenced by both anthropogenic and natural drivers such as climate change, rapid urbanization, and widespread travel. Over time, the unprecedented rise of new and re-emerging zoonotic diseases has prompted the need for rapid and effective vaccine development. Following the success of the COVID-19 mRNA vaccines, mRNA-based platforms hold great promise due to their rapid design, swift development and ability to elicit robust immune responses, thereby highlighting their potential in combating emerging and pre-pandemic zoonotic viruses. In recent years, several mRNA vaccines targeting emerging and re-emerging zoonotic viral diseases, such as rabies, Nipah, Zika, and influenza, have advanced to clinical trials, demonstrating promising immunogenicity. This review explores recent advances, challenges, and future directions in developing mRNA vaccines against emerging and re-emerging zoonotic viral diseases.

## 1. Introduction

Zoonotic diseases, or zoonoses, are infectious diseases that are transmitted from vertebrate animals to humans during spillover events [1,2]. Zoonoses cause millions of deaths and approximately one billion cases of human illness annually [3]. These diseases are caused by a range of pathogens, which include bacteria, viruses, parasites, and fungi. It is estimated that 60.3% of the emerging infectious diseases are caused by zoonotic pathogens, with 71.8% of these attributed to pathogens of wildlife origin, such as the emergence of the Nipah virus from Malaysia [4]. An epidemiological study identified 1415 pathogens that are known to cause disease in humans, revealing that 61% (868) were zoonotic, whilst 175 zoonotic pathogens were associated with emerging infectious diseases [5]. Zoonoses can be widely transmitted through several routes, including inhalation of airborne pathogens, direct contact with infected animals, and vector-borne transmission via mosquitoes, fleas, and ticks [6]. For instance, the Rift Valley fever virus (RVFV) is transmitted via direct or indirect contact with the blood or organs of infected animals and humans, whilst the Seoul virus is primarily transmitted via inhalation of aerosolized urine or saliva from infected black rats.

The emergence and re-emergence of zoonoses are driven interchangeably by several factors such as massive population growth, socio-cultural behaviors, agricultural practices, and unprecedented climate change, which contribute to an increase in human–wildlife interactions and the rapid spread of zoonotic pathogens [7]. For instance, the Southeast Asian (SEA) region is an example of a major hotspot for zoonotic diseases. First, the population of the SEA region increased significantly by 47% from 1980 to 2022, enabling rapid disease spread due to high human density [8]. With tourism being a major source of income for countries in the SEA region, enhanced accessibility to countries enables the spread of diseases and the introduction of vectors into new environments, which increases the risk of new outbreaks [9]. Second, habitat destruction, mainly attributed to deforestation, increases human–wildlife interactions and fosters vector population dynamics that promote transmission, facilitating zoonotic spillover events [8]. Third, agricultural practices such as the illegal trade of wildlife, the availability of traditional live animal markets, and intensive farming exacerbate the risk of zoonotic transmission. These practices are responsible for the loss of biodiversity, increased water demand, and further enhanced the spillover of pathogens via the confinement of livestock in stressful conditions and the consumption of wild meat [10]. Lastly, unprecedented climate change contributes to the gradual rise in zoonotic diseases by influencing the transmission and distribution dynamics of pathogens and vector-borne diseases [11]. In the case of vector-borne zoonotic diseases, rising global temperature promotes the acceleration of the life cycles of arthropod vectors such as mosquitoes, leading to the rapid reproduction of mosquitoes.

## 2. Vaccines: From Discovery to Global Utilization

A preventive vaccine against infectious disease is a biological product that, when introduced into the body of the vaccinated human or animal, facilitates the acquisition of immunity to a specific infectious disease [12]. Over the past century, vaccination has stood out as one of the most effective public health interventions, significantly reducing illness and infection-related deaths [13]. The World Health Organization (WHO) estimates that preventive vaccines can save approximately 2–3 million lives annually worldwide. While inoculation practices dated back over 500 years, the term “vaccine” was first coined by Edward Jenner in the 18th century, deriving from the Latin word, vacca (cow) [14]. In 1796, Jenner inoculated an eight-year-old boy with material from cowpox lesions which were obtained from a milkmaid’s hand and successfully demonstrated the protection against smallpox. This significant breakthrough laid the foundation for modern vaccination. Through a coordinated global effort led by the WHO, smallpox became the first and remained the only human infectious disease that was completely eradicated by 1980. Thus, the first golden age of vaccines emerged with the establishment of the germ theory, which states that specific microorganisms known as germs or pathogens are the cause of specific diseases [13,15]. This led to the development of the first generation of vaccines, which were based on live-attenuated or inactivated (killed) pathogens as well as inactivated toxins (toxoids), which successfully protected humans against cholera, rabies, tuberculosis, and tetanus [16,17]. During the second half of the 20th century, major advances in cell culturing techniques paved the way for the second golden age of vaccines, leading to the successful development of major live-attenuated vaccines against polio, measles, mumps, rubella (MMR), and varicella [17,18,19]. Advances in microbiological research gave rise to the development of polysaccharide vaccines against infectious diseases caused by bacteria such as pneumococcus, *Haemophilus influenzae*, and meningococcus types (A, C, W, and Y) [13]. The introduction of recombinant DNA and genomic approaches like reverse vaccinology and genomic sequencing significantly contributed to the development of vaccines against human papillomaviruses (HPV), hepatitis B virus (HBV), and *Neisseria meningitidis* type B (MenB).

Traditional vaccines work on the principle of eliciting protective immunity by exposing the immune system to a harmless version or components of a pathogen, thereby priming the body to recognize and combat future infections caused by the pathogen. Traditional vaccines are categorized by two key criteria: their capacity for replication within the host (e.g., live attenuated versus inactivated vaccines) and the technological platforms employed in their production [20]. Live-attenuated vaccines utilize weakened pathogens that retain the ability to replicate in the host, mimicking natural infection without causing disease. An important feature of this platform involves maintaining replication competence while minimizing the risk of reversion to virulence. Inactivated vaccines utilize chemically killed pathogens to stimulate antibody-mediated immunity, eliminating the risk of replication or virulence reversion. While they offer advantages like low-cost production and dispense with the requirement for ultra-low temperature storage, they often require adjuvants due to weaker immunogenicity compared to live vaccines. On the other hand, subunit vaccines utilize purified pathogen components like proteins or polysaccharides to safely elicit targeted immune responses, making them suitable for immunocompromised individuals [21]. Subunit vaccines often require complex time-consuming manufacturing and additional adjuvants due to limited immunogenicity.

Conventional vaccine platforms face limitations in rapidly addressing emerging pandemic threats due to manufacturing constraints. This has driven the development of adaptable nucleic-acid-based technologies, starting with the recombinant DNA vaccine, evolving through the utilization of viral vectors, including adeno-associated virus (AAV) or adenoviral vectors and, most recently, through the introduction of mRNA vaccines [22]. These advances demonstrated that minimal viral protein components could induce robust protective immunity, revolutionizing vaccine design by rapid and precise targeting of emerging viruses. Thus, the rapid development and swift approval of messenger RNA (mRNA) vaccines during the recent COVID-19 pandemic is considered one of the most significant milestones in vaccine development history [23]. The development of the COVID-19 mRNA vaccines was made highly possible by the foundational mRNA research that had been strongly established approximately three decades ago [23,24,25]. The first successful utilization of in vitro transcribed (IVT) mRNA in animals was documented in 1990, when mRNAs for reporter genes were injected into mouse skeletal muscles in vivo, resulting in protein expression being detected via luciferase reporter activity [26]. A subsequent study in 1992 showed that the administration of synthetic vasopressin- encoding mRNA into rats genetically deficient in vasopressin successfully induced the temporary reversal of diabetes insipidus [27]. In 1993, another study demonstrated that the liposome-entrapped IVT encoding the influenza virus nucleoprotein (NP) successfully elicited adaptive immune responses in mice which closely resembled the immune responses of virus-infected mice [28]. Despite these early promising findings, significant progress in the development of mRNA therapeutics and vaccines remained limited, primarily due to several underlying concerns about the structural instability, inefficient in vivo delivery, and immunogenicity of the mRNA. However, the efficacy and potential of the mRNA vaccine technology were fully realized when the COVID-19 mRNA vaccines were successfully developed by Pfizer-BioNTech and Moderna [23].

## 3. Advances of mRNA Vaccines

Over the last decade, major research investments and technological innovations have primarily driven extensive research into improving and enhancing the overall mRNA features and quality [24]. Key mRNA features targeted for improvement include delivery through encapsulation in lipid nanoparticles (LNPs), reduction of innate immune responses against RNA through the incorporation of modified nucleotides, enhanced stability via introduction of point mutations, 5′ capping, 3′ tailing as well as increased purity to reduce unwanted innate immune responses to enable efficient protein expression [23]. mRNA vaccines offer several advantages over traditionally established live-attenuated virus and subunit vaccines. First, mRNA utilizes human host cells as its vaccine-production facility [29]. Unlike conventional vaccines, which rely heavily on bulk production using chicken eggs or mammalian cells in bioreactors, mRNA vaccines are translated into final protein products directly within the host cells after administration. Second, mRNA vaccines can be scalable, rapidly and inexpensively manufactured in a cell-free environment [30]. Batches of the vaccine can be rapidly generated following the availability of the sequence encoding the immunogen of interest. Facilities specifically dedicated to mRNA production can rapidly manufacture vaccines against multiple targets of interest, ideally minimizing changes to the formulation and processes. Third, the currently licensed COVID-19 mRNA vaccines are clinically safe, as they have a favorable benefit–risk ratio and potentially vaccine-specific safety issues seem to mainly occur in younger men [24,31]. Given that mRNA encoding a single antigen has the clear advantage of not being able to produce infectious particles or DNA by reverse transcription, there is no likely risk of infection or unforeseen insertional mutagenesis caused by integration into the host DNA. Lastly, mRNA vaccines exhibit vaccine immunogenicity, as several modifications within the mRNA structure will enhance mRNA stability, translation efficiency, and reduce innate immune activation [23,24]. Highly efficient in vivo delivery can be accomplished by formulating mRNA into carrier molecules like non-lipid polymers and ionizable LNPs, which can protect mRNA from rapid degradation and facilitate delivery into the cytoplasm with minimal toxicity [32].

**Figure 1:**
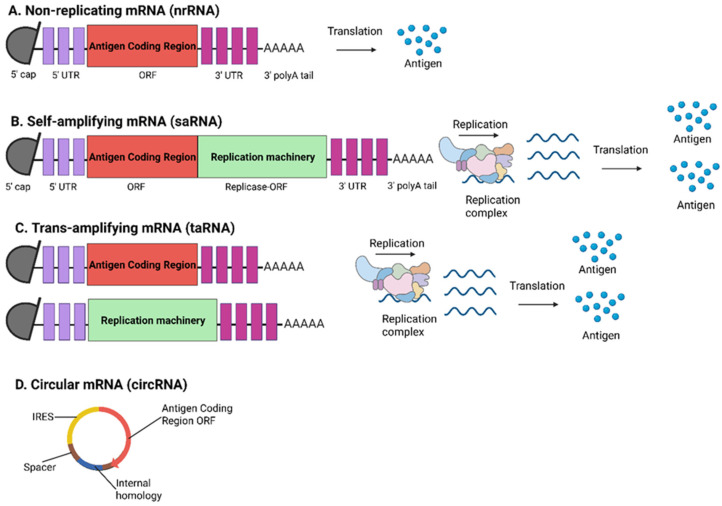
Different types of mRNA vaccines. (**A**) Non-replicating mRNA (nrRNA) contains a 5′ cap, 5′ and 3′ UTRs, antigen coding region (ORF), 3′ UTR, and a 3′ poly(A)tail. (**B**) The self-amplifying mRNA (saRNA) features all mRNA components of nrRNA and an additional RNA replication machinery. (**C**) The trans-amplifying mRNA (taRNA) is composed of two individual mRNAs: one encoding the antigen coding region, whilst the other contains the RNA replication machinery. (**D**) The circular mRNA (circRNA) contains an internal ribosome entry site (IRES) element, ORF, and the internal homology region. The figure was created in https://BioRender.com.

mRNA vaccines can be broadly categorized into two main types based on their mechanism of action: non-replicating mRNA (nrRNA) that encodes the target antigen only (Figure 1A) and the self-amplifying mRNA (saRNA) that encodes both the target antigen and viral replication proteins, allowing the mRNA to replicate within transiently transfected cells (Figure 1B) [33]. More recently, two additional types of mRNA have been added to the classification: trans-amplifying mRNA (taRNA) (Figure 1C) and circular mRNA (circRNA) (Figure 1D) [34]. Like natural mRNA, the general structure of nrRNA consists of five elements: a 5′ cap, a 5′ untranslated region (UTR), an open reading frame (ORF) encoding the gene of interest, and a 3′ UTR, and a poly(A) tail. The advantage of nrRNA vaccines includes a simple and well-validated design, enabling immediate utilization of the vaccine following IVT. However, as nrRNA is unable to replicate in vivo, higher doses are often needed to elicit an effective immune response. Prominent examples of nrRNA vaccines are the COVID-19 vaccines utilized during the COVID-19 pandemic, BNT162b2 and mRNA-1273, manufactured by Pfizer-BioNTech and Moderna, respectively [35]. saRNA vaccines are derived from engineered mRNA based on single-stranded RNA virus genomes such as flaviviruses and alphaviruses [36]. These vaccines incorporate the target gene of interest in place of viral structural proteins while retaining the non-structural gene encoding the viral RNA replication machinery. This enables mRNA to self-amplify within the host, leading to significantly higher antigen expression and immune responses, even at minimal doses [34]. As saRNA transcripts are considerably longer and more complex due to the inclusion of the self-amplifying elements, these pose underlying challenges in both manufacturing and delivery, in terms of efficient encapsulation of the saRNA.

Utilizing a novel bipartite vector system, the taRNA vaccine provides broader applicability, a simplified design for easier production, and an enhanced safety profile [37]. Unlike saRNA vaccine design, which combines both the target gene of interest and the self-replicating elements within a single mRNA transcript, tRNA separates these components: the target genes required for antigen expression are encoded in one RNA strand, whilst the self-replicating RNA machinery is provided via a separate cassette [38]. By decoupling these components, taRNA reduces the overall molecular length and provides an edge in manufacturing scalability, delivery efficiency, and formulation versatility over other mRNA types. As shorter mRNA transcripts are generally more straightforward, taRNA vaccines offer long-term cost-effective potential without compromising high yield and quality. Separating the antigen-encoding sequences from the self-amplification elements reduces the risk of generating replication-competent RNA viruses [34]. CircRNAs are covalently closed, circular biomolecules known for their extended half-life, which shields the RNA from environmental degradation [39]. In vitro transcribed circRNA normally utilizes an intron-splicing approach to facilitate the circularization of the target RNA [28]. Thus, this novel system offers significant advantages in functionality and stability, making circRNA a promising platform for mRNA vaccine development and broader therapeutic applications.

## 4. Optimization Approaches for mRNA Vaccines

**Figure 2:**
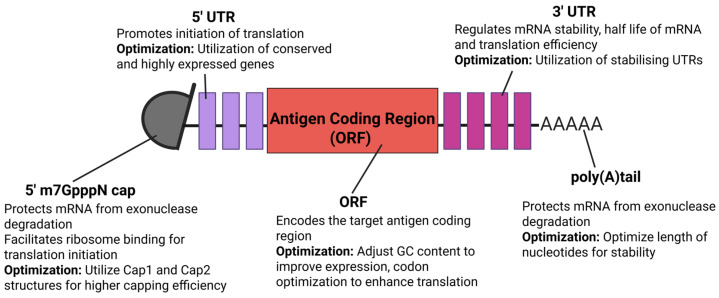
Structural features of in vitro transcribed (IVT) mRNA for optimized stability and translation efficiency. The figure was created in https://BioRender.com.

The efficacy of mRNA vaccines depends critically on strategically optimizing molecular structure to enhance translational efficiency, stability and immunogenicity. Several notable strategies include refining the 5′ cap structure, engineering untranslated regions (UTRs), nucleoside modifications, and even LNP encapsulation. Together, these approaches highlight the potential of mRNA vaccine technology, enabling rapid design against emerging and re-emerging zoonotic viral pathogens without compromising immunogenicity and safety (Figure 2) [40]. The 5′ cap is a modified nucleotide structure that comprises a 7-methylguanosine (m7G) linked to the first nucleotide of the mRNA via a 5′-5′ triphosphate bridge (m7GpppN). The inclusion of a 5′ cap structure is essential for mRNA stability and functions [41]. The 5′ cap facilitates recruitment of ribosomes through interaction with the eukaryotic translation initiation factor (eIF4E), enhances mRNA translation efficiency, and protects the mRNA from 5′ degradation catalyzed by 5′ exonucleases, thus extending the half-life of mRNA transcripts in the cytoplasm. Incorporation of the 5′ cap can be achieved via a post-transcriptional enzymatic reaction, which involves the utilization of vaccinia-virus capping enzyme or *Faustovirus* capping enzyme to cap the uncapped mRNA following the generation of newly synthesized IVT mRNA. On the other hand, the co-transcriptional capping involves the addition of a cap analog into the IVT reaction mixture, thus automatically enabling the 5′ cap to be integrated into nascent mRNA transcripts and eliminating the need for further enzymatic reaction steps. Additionally, post-translational modification such as 2′-O methylation, which involves converting the 5′ cap-0 structure to a 5′ cap-1 structure within the nascent IVT mRNA, has been demonstrated to suppress innate immunogenicity and enhance translation efficiency [38].

Located at both the 5′ and 3′ ends of the mRNA, the untranslated regions (UTRs) are involved in translation efficiency, regulating mRNA stability and subcellular localization by interacting with species-specific RNA-binding proteins [41]. In eukaryotes, the 5′ UTR normally contains 100 or more nucleotides, whilst the 3′ UTR is generally longer, often extending to several kilobases [42]. On average, 3′ UTRs are about twice as long as 5′ UTRs. The 5′ UTR possesses upstream open reading frames (uORFs) and stabilizes secondary structures like hairpins, which are essential in regulating translation efficiency [35]. The 5′ UTR normally promotes the initiation of translation, whilst the 3′ UTR is more closely associated with regulating the stability and half-life of mRNAs [40]. To enhance 5′ UTR translation efficiency, sequences can be engineered from a variety of genes such as hydroxysteroid dehydrogenase (3β-HSD), Hsp70, and globin. To improve mRNA stability, a 3′ UTR can be engineered from genes such as albumin (ALB), hemoglobin subunit α (HBA), and subunit β (HBB) genes. Interestingly, both 5′ and 3′ UTRs derived from one of the most efficiently expressed mammalian mRNAs, human hemoglobin subunit β (hHBB), have demonstrated increased mRNA stability and enhanced protein production [38]. Thus, the 5′ UTR can be engineered to improve translation efficiency while the 3′ UTR can be optimized to stabilize mRNA and increase protein expression, ultimately improving the immunogenicity of mRNA vaccines.

The open reading frame (ORF) of mRNA is an essential region that encodes the target protein, starting with a start codon and ending with a stop codon. Being flanked by the 5′ and 3′ UTRs, the design of ORF is important, as it directly involves the production of the target antigen. The sequence and length of the ORF determine the identity and structure of the synthesized protein [41]. In general, ORF sequences are thoroughly optimized to improve mRNA stability and translation efficiency. One commonly utilized approach is codon optimization, which significantly improves translation efficiency and reduces the likelihood of premature termination. Another approach involves incorporating specific RNA modifications to enhance mRNA stability and promote accurate translation. Modifications of nucleotides such as N1-methyl pseudouridine and 5′methylcytosine have been demonstrated to increase translational efficiency, thus contributing to the overall immunogenicity of mRNA vaccines [40]. Additionally, adjusting the GC content of mRNA improves translation efficiency and stability, as high GC content promotes the formation of stable secondary structures that shield the mRNA from degradation. However, excessively high GC content may introduce overly strong secondary structures that interfere with translation elongation. By optimizing GC content, codon usage, and uridine reduction, a comprehensively modified ORF can effectively support high levels of protein expression whilst reducing the risk of innate immune system activation, which are key factors for the success of mRNA vaccines.

The poly(A) tail is a stretch of 50 to 250 adenine (A) nucleotides added to the 3′ end of the eukaryotic mRNA transcripts during transcription. It is essential for nuclear export of mRNA to the cytosol, mRNA stability, and translation efficiency [41]. The length of the poly(A) tail must be thoroughly optimized, as shorter poly(A) tails are associated with rapid mRNA degradation and decreased translation efficiency, whilst excessively long poly(A) tails can reduce translation efficiency by altering RNA-protein interactions. The poly(A) tail can be added to mRNA transcripts either through a post-transcriptional, enzyme-mediated process using poly(A) polymerase or through direct synthesis during the IVT reaction [38].

## 5. mRNA Vaccines Against Emerging and Re-Emerging Viral Zoonoses

Emerging and re-emerging zoonotic diseases are increasingly posing significant threats to global public health, as they have the potential to cause large outbreaks and pandemics. These zoonoses have been comprehensively studied for many centuries, and efforts to develop vaccines against them are potentially regarded as some of the most remarkable achievements in vaccinology. In 2019, the Centers for Disease Control and Prevention (CDC) in the USA identified the top eight zoonoses of greatest national concern in the US, which were zoonotic influenza, salmonellosis, plague, West Nile virus, emerging coronaviruses, rabies, brucellosis, and Lyme disease [43]. Among these, four are classified as zoonotic viral diseases. The recent success of mRNA vaccines against SARS-CoV-2 (COVID-19) during the COVID-19 pandemic has subsequently paved the way for their applications against other zoonotic threats posed by Ebola virus, Nipah virus, Zika virus, Crimean-Congo hemorrhagic fever (CCHF) virus, rabies virus, West Nile virus, avian influenza virus, and Lassa virus. Although COVID-19 mRNA vaccines remain the only licensed mRNA vaccines to date, numerous vaccine candidates targeting other zoonoses are currently in various stages of preclinical and clinical development. Here, we evaluate recent advances in mRNA vaccine development and their potential to address zoonotic viral diseases, while highlighting the challenges associated with their implementation.

### 5.1. mRNA Vaccines for Coronaviruses

Historically, coronaviruses (CoVs) pose a significant threat to humans and animals, with several CoV-related outbreaks causing severe human diseases. Between 2002 to 2003, severe acute respiratory syndrome coronavirus (SARS-CoV) infected over 8000 individuals globally with approximately 800 fatalities, resulting in a 10% fatality rate [44]. A decade later, in 2012, Middle East respiratory syndrome coronavirus (MERS-CoV) was first identified in Saudi Arabia, and infected over 857 individuals, resulting in 334 deaths, a staggering mortality rate of 35% [45,46]. Porcine Epidemic Diarrhea Coronavirus (PED), an Alphacoronavirus within the *Coronaviridae* family, rapidly emerged in the United States, Canada, and Mexico in 2013, killing more than 8 million newborn piglets and decimating 10% of the U.S. swine population [47,48]. In late 2019, SARS-CoV-2 first emerged in Wuhan, China, which rapidly evolved to cause a global pandemic due to its high transmissibility, primarily through respiratory droplets and aerosols spread via coughs and sneezes [49]. By the end of 2023, there were over 772 million confirmed cases of SARS-CoV-2, with nearly seven million deaths globally [50,51].

**Figure 3:**
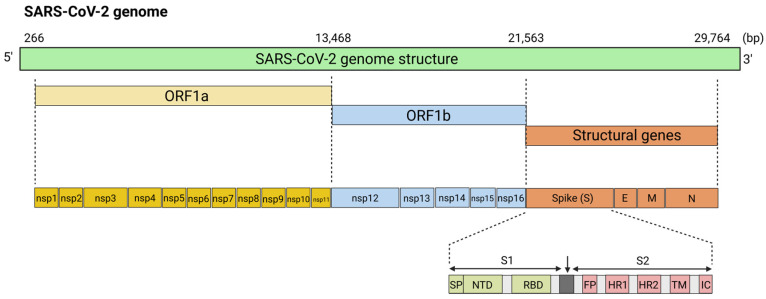
Schematic diagram of the SARS-CoV-2 genome structure. The SARS-CoV-2 genome contains two large genes, ORF1a (yellow) and ORF1b (blue), which encode 11 (nsp1–nsp11) and 5 (nsp12–nsp16) non-structural proteins, respectively. The structural genes (orange) encode the structural proteins, spike (S), envelope (E), membrane (M), and nucleocapsid (N). The spike glycoprotein consists of receptor-binding S1 (green) and membrane-fusion S2 (red) subunits. SARS-CoV-2 spike is proteolytically activated at the S1/S2 boundary (black), where S1 dissociates and S2 undergoes a distinct structural change. S1 constitutes the signal peptide (SP), N-terminal domain (NTD), and the receptor-binding domain (RBD). S2 constitutes the fusion peptide (FP), two heptad-repeat domains (HR1 and HR2), a transmembrane domain (TM), and a C-terminal intracellular tail (IC). The figure was created in https://BioRender.com.

Coronaviruses are large positive-sense RNA viruses measuring approximately 60–140 nm in diameter [52]. The viral genome is encapsulated within a symmetrical helical nucleocapsid composed of nucleocapsid (N) proteins, which are enclosed by an outer envelope [53]. This viral lipid bilayer envelope is composed of three structural proteins: the membrane protein (M) and the envelope protein (E), which are involved in virus assembly, and the spike protein (S), which facilitates host cell entry. The S protein forms distinctive spike-like projections on the virus surface, which create a “crown-like” appearance when observed under the electron microscope, hence earning the name coronaviruses [54]. The genomic structure of CoVs encodes two large genes, ORF1a and ORF1b, which encode 16 non-structural proteins (nsp1-nsp16) that are involved in viral replication (Figure 3) [55]. The S protein is composed of three structural segments: a large ectodomain, a single-pass transmembrane anchor, and a short intracellular tail [53]. The ectodomain is divided into two functional subunits, S1, which is responsible for receptor binding, and S2, which is involved in mediating the fusion of the viral cell membrane with the host [56]. The S1 protein is a critical component of infection, and it consists of two domains, the N-terminal domain (S1-NTD) and the C-terminal receptor-binding domain (S1-RBD) [57]. The S2 protein contains a fusion peptide (FP) domain, two heptad-repeat domains (HR1 and HR2), a transmembrane anchor (TM), and a C-terminal intracellular tail (IC) [58]. The S1 protein attaches to a host cell receptor, angiotensin-converting enzyme 2 (ACE2), which facilitates viral entry into the host cell [59]. When S protein binds to the receptor, transmembrane protease serine 2 (TMPRSS2) primes the S protein for membrane fusion, enabling the virus to enter the host cells efficiently. Following entry, viral RNA is released and translated into polyproteins by the host cell machinery. Newly generated polyproteins are then cleaved to form the replicase-transcriptase complex (RTC), which is essential for viral replication and transcription. Nascent viral RNA is replicated, and structural proteins are synthesized, assembled, and packaged into progeny viruses before they are released from the host cell via exocytosis.

Initially, MERS-CoV has been suggested to be transmitted from bats [60]. However, as human–bat contact is relatively limited, dromedary camels have been implicated to be the probable reservoir host for MERS-CoV, although the nature of infection from camels is not well-established [61]. Although respiratory transmission is believed to be the most likely route of MERS-CoV transmission, camel milk has also been investigated as a potential transmission route, given that it is common to consume camel milk in the Arabian Peninsula [60]. Almost all the current vaccine candidates for MERS-CoV are still in development, and none have yet been approved for human use. Most of the vaccine candidates are based on currently available vaccine platforms such as DNA (GLS-5300) [62], protein nanoparticle-based vaccine (MERS-CoV RBD) [63], viral vectored (ChADOx1-MERS and MVA-MERS-S) [64], and recombinant protein vaccine candidate (LV-MS1-Fc) [65], all of which are still in pre-clinical or clinical stages of development. Although there are very minimal advancements towards the development of a MERS-CoV mRNA vaccine, there are suggestions to incorporate the RBD of the MERS-CoV S protein as an antigen due to its ability to elicit potent neutralizing antibodies [66]. Additionally, preclinical studies in mice demonstrated that pseudouridine-modified RBD-mRNA vaccine successfully induced adaptive immune responses, which include germinal center B cells and T follicular helper cells, while minimizing the production of inflammatory cytokines. Further studies showed that mice vaccinated with the pseudouridine-modified RBD mRNA vaccine encapsulated in LNPs elicited potent neutralizing antibodies for at least 6 months and broad cross-neutralization against 10 MERS-CoV variant strains when compared to the unmodified RBD-mRNA-LNP wild type vaccine candidate [67]. Mice vaccinated with the modified RBD mRNA vaccine were completely protected against challenge with live MERS-CoV and had undetectable lung viral loads, demonstrating its potential as an effective and safe MERS-CoV vaccine candidate.

During the COVID-19 pandemic, several routes of transmission of SARS-CoV-2 have been potentially considered, including airborne, bloodborne, via fomites, animal to human, contact, droplet-mediated, and even mother to child transmission [68]. Early observations during the pandemic suggested that being close to an infected person plays a role in the risk of transmission [69]. For instance, a comprehensive contact tracing study on the Diamond Princess outbreak revealed that SARS-CoV-2 spread either through close contact in public areas or between passengers sharing cabins with an infected passenger [70]. Later on, SARS-CoV-2 was identified to predominantly spread through respiratory transmission via droplets and aerosols [69]. The U.S. Food and Drug Administration (FDA) granted two mRNA-based vaccines for Emergency Use Authorizations (EUA) [71]. The Pfizer-BioNTech BNT162b2 (Cominarty) and Moderna mRNA-1273 (Spikevax) were licensed for vaccination in December 2020 and January 2021, respectively. Both vaccines are nucleoside-modified mRNA that were encapsulated and delivered via LNPs. The BNT162b2 vaccine contains an mRNA encoding the full-length S protein of SARS-CoV-2 with two proline substitutions (amino acid positions 986 and 987) within the S2 to stabilize the S protein in the pre-fusion conformation [72]. On the other hand, the mRNA-1273 vaccine carried an mRNA encoding the full-length S glycoprotein with two proline substitutions within the S2 and an intact furin cleavage site. Approved dosage regimens included two primary doses, with up to three doses for the general population and four for high-risk groups [73]. Globally, hundreds of millions of mRNA vaccine doses have been administered since the start of the COVID-19 pandemic, which has enabled the consolidation of the efficacy and safety data on these vaccines.

### 5.2. mRNA Vaccines for Ebolavirus (EBOV)

**Figure 4:**
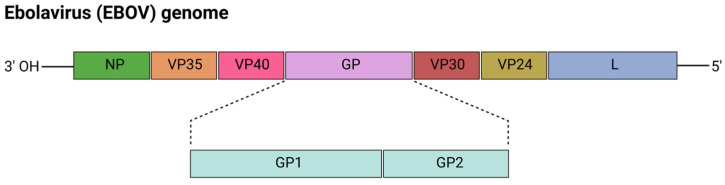
Schematic diagram of the Ebolavirus (EBOV) genome structure. The single-stranded negative-sense RNA EBOV genome encodes seven proteins (NP, VP35, VP40, GP, VP30, VP24, L) flanked by 3′ and 5′ untranslated regions. The surface glycoprotein (GP) forms trimers and is composed of GP1 and GP2 subunits linked by a disulfide bond. The figure was created in https://BioRender.com.

Ebolaviruses (EBOV) are negative-sense RNA viruses that belong to the genus *Ebolavirus* of the *Filoviridae* family and are highly endemic to parts of West and equatorial Africa (Figure 4) [74]. The *Ebolavirus* genus comprises five recognized species: Bundibugyo virus, Ebola virus, Reston virus, Sudan virus, and Tai Forest virus. So far, only the Bundibugyo, Ebola, and the Sudan viruses have predominantly caused outbreaks in humans in the Democratic Republic of the Congo, Gabon, the Republic of Congo, South Sudan, and Uganda. The Ebola virus, which has a fatality rate of 80–90%, caused the largest recorded outbreak of Ebola virus disease (EVD) in West Africa from 2014–2016 [75]. The outbreak was officially declared to be over in June 2016 by the WHO, after more than 28,600 individuals had been infected and 11,325 infected persons had died. In September 2022, Uganda officially declared a Sudan virus-associated EVD outbreak, which affected 164 individuals and resulted in 55 fatalities [76]. Ebola virus disease is considered to be zoonotic as current evidence suggests that fruit bats of the *Pteropodidae* family serve as natural reservoirs [77]. EVD outbreaks have been greatly associated with zoonotic infections, migration of bats, and spillover events. Humans are unintentionally being infected through the handling of infected animals or direct/indirect contact with infected bats. Secondary human-to-human transmission typically occurs via inoculation of the virus into the bloodstream or exposure to infected body fluids and secretions, such as blood from infected humans. Handling deceased infected individuals during traditional funerals or even caregiving for infected individuals increases the risk of Ebola transmission [78]. As such, these transmission pathways significantly contributed to nosocomial spread, particularly at the start of an outbreak [74]. Ebola virus pathogenesis is characterized by widespread systemic infection targeting multiple cell types, particularly dendritic cells which drive multifaceted pathogenesis [77]. This involves massive immune dysregulation characterized by immune hyperactivation, immune suppression, cytopathic effects, and tissue damage. Thus, in the absence of intensive supportive care, these cascading effects progress to multi-organ failure and death within 10 days of symptom onset in humans.

Several EBOV vaccines, including the live-attenuated recombinant vesicular stomatitis vaccine (rVSV) (ERVEBO^®^ by Merck, Darmstadt, Germany) and the recombinant Ad26.ZEBOV (Zabdeno)-MVA-BN-Filo (Mvabea) vaccine manufactured by Johnson & Johnson (New Brunswick, NJ, USA)/Janssen (Beerse, Belgium), were licensed for human use [79]. However, challenges such as cold-chain requirements, reactogenicity, undefined vaccine efficacy, and short-lived immunity necessitated the need for boosters. Meyer and colleagues (2018) developed two novel nucleoside-modified mRNA vaccine candidates encoding Ebola virus glycoprotein (GP) with different signal peptides which were encapsulated in LNPs [80]. Both vaccine candidates successfully elicited neutralizing antibodies, with Vaccine B containing the human kappa immunoglobulin signal peptide capable of inducing higher GP-specific IgG and neutralizing antibodies when compared to Vaccine A, which carried the wild type Ebola virus GP signal peptide. Both Vaccine A and Vaccine B candidates successfully protected immunized guinea pigs after being challenged with lethal guinea pig-adapted Ebola virus. This in vivo study demonstrated the utilization of novel mRNA-LNP vaccines against EBOV and represented a promising alternative to current viral- vectored Ebola virus vaccines, with strong efficacy and immunogenicity being demonstrated in vivo. The authors suggested the need for further testing of these mRNA-LNP vaccine candidates in nonhuman primates as a critical step toward clinical testing.

### 5.3. mRNA Vaccines for Nipah Virus (NiV)

Nipah virus (NiV) is a highly pathogenic zoonotic RNA virus belonging to the *Henipavirus* genus, which also consisted of Hendra virus and Cedar virus of the *Paramyxoviridae* family [81]. The first few cases of NiV infection were identified during the 1998–1999 NiV outbreak in peninsular Malaysia, with 265 cases of acute encephalitis resulting in 105 deaths and the near collapse of Malaysia’s pig-farming industry due to the culling of over a million pigs [82]. Due to the import of pigs from Malaysian farms, the NiV outbreak affected Singapore, which led to 11 infection cases, with one fatality among abattoir workers. Following the discovery of the virus in 1999, fruit bats of the *Pteropodidae* family were identified as the natural host reservoir for NiV [83]. The transmission of NiV to pigs likely occurred when these fruit bats contaminated partially eaten fruits with saliva or urine, which unintentionally fell into pig enclosures and subsequently infected pigs that consumed the contaminated fruits. The most prominent hotspot for NiV zoonotic transmission is Bangladesh, which experienced annual NiV outbreaks, demonstrating repeated zoonotic spillover events from bats to humans [84,85]. NiV might pose a geographical threat as the virus has been detected in *Pteropus* bats and other bat species across several countries, including Cambodia, Ghana, Indonesia, Madagascar, the Philippines, and Thailand [86]. These findings highlighted the potential for NiV to emerge in new regions, signaling significant public health risks. NiV in humans causes a range of clinical manifestations, from being asymptomatic to fatal encephalitis, with fatality rates ranging from 40% to 75%.

**Figure 5:**
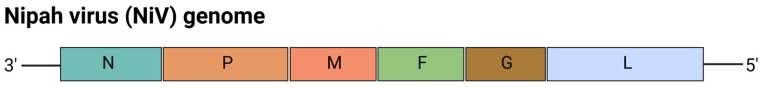
Schematic diagram of the Nipah virus (NiV) genome structure. NiV genome is a single-stranded, negative-sense RNA encoding six structural proteins (N, P, M, F, G, L), which are arranged accordingly from 3′ to 5′. The figure was created in https://BioRender.com.

NiV is a negative-sense single-stranded RNA virus carrying a non-segmented 18kb viral genome encoding six structural proteins: fusion glycoprotein (F), attachment glycoprotein (G), matrix protein (M), nucleoprotein (N), phosphoprotein (P), and the RNA polymerase large protein (L) (Figure 5) [87]. The two NiV surface glycoproteins, the G protein which mediates host-cell entry, and the F protein, which mediates membrane fusion, are recognized as key antigenic binding sites for neutralizing antibodies, thus making them primary targets for NiV vaccine candidates [88]. Given NiV’s high case mortality rate, developing a safe live-attenuated vaccine without any reversion risk is particularly challenging [87]. As a result, most of the NiV vaccine candidates are developed on other vaccine platforms such as subunit vaccines and viral-vectored vaccines. As of 2025, several NiV vaccine candidates, such as the HeVsG recombinant subunit vaccine that was originally developed for Hendra virus and the ChAdOx-1- vectored vaccine, appeared to be promising and are in the pre-clinical development stage [89,90]. Apart from these vaccine platforms, there are several NiV mRNA-based vaccine candidates currently under development. Lo et al. (2020) evaluated a nucleoside-modified mRNA-LNP vaccine candidate encoding the soluble Hendra virus glycoprotein (sHeVG) in immunized Syrian hamsters against the lethal NiV challenge [91]. A single dose of the mRNA-LNP vaccine demonstrated partial protection up to 70% in hamsters against a lethal NiV challenge. Despite the surviving hamsters displaying reasonable post-challenge immune responses, such as the elicitation of NiV-specific IgG and neutralizing antibodies, the authors highlighted the need to optimize the vaccination dose and route to demonstrate the potential of an mRNA-LNP vaccine against NiV.

Loomis et al. (2021) developed several nucleoside-modified mRNA-LNP vaccine candidates encoding a combination of NiV antigens, including the chimeric NiV F/G to evaluate the route of mRNA delivery and immunogenicity in CB6F1/J mice [92]. The mRNA-LNP vaccine encoding the prefusion-stabilized fusion glycoprotein (pre-F) + G interestingly elicited strong neutralizing antibodies and more robust T-cell immune responses when compared to the other mRNA-LNP vaccine candidates encoding the other antigens (pre-F only, post-F only, Hex G only). Thus, this proof-of-concept study demonstrated the potential of a NiV vaccine candidate based on an mRNA platform that could be further advanced to pre-clinical and clinical stages. A related study evaluated the immunogenicity of a nucleoside-modified mRNA-LNP vaccine candidate encoding the soluble glycoprotein (sG) from the Malaysian NiV strain (NiV-M) isolated from pigs, given their roles as intermediate NiV hosts [93]. Two doses of the vaccine elicited potent antigen binding and virus-neutralizing antibodies, which mediated effective neutralization of both NiV-M and Bangladesh NiV (NiV-B) strains but displayed minimal cross-neutralizing activity against Hendra virus. Following booster immunizations, the NiV-sG vaccine candidate induced both CD4+ and CD8+ T-cell responses, supporting further development of this mRNA-LNP vaccine in other pre-clinical animal models and eventually in humans. Most recently, Brandys and colleagues (2024) reported the development of a NiV mRNA vaccine candidate comprising a 60-mer nanoparticle (NP) displaying 60 head domains of NiV glycoprotein (mRNA NiV G-NP) as a proposed novel vaccine candidate against NiV [94]. A single dose of the mRNA NiV G-NP vaccine candidate successfully elicited a robust humoral response and NiV neutralizing antibody response in immunized-C6BF1/J mice, highlighting the potential utilization of NiV mRNA-NP vaccines for emergency use during future NiV outbreaks, although further evaluations of this vaccine candidate remain a priority. Additionally, Moderna’s NiV vaccine candidate (mRNA-1215), which encodes the (pre-F/G) of the NiV-M strain, has entered phase I clinical trial, which evaluated the safety and the immunogenicity of the mRNA-1215 vaccine candidate in healthy participants [95]. Currently, there are no updates on the outcome of the mRNA-1215 phase I clinical trial (NCT05398796).

### 5.4. mRNA Vaccines for Influenza Virus

Influenza is a highly contagious respiratory disease caused by influenza viruses that circulate in a considerable number of animal species, including birds and pigs. Seasonal influenza causes one billion infections annually, with 3 to 5 million infections progressing to severe illness and resulting in 290,000 to 650,000 deaths globally [96]. Approximately 99% of deaths occurred in children under five in developing countries. There are four types of influenza viruses: A, B, C, and D. Influenza A (IAV) and B (IBV) viruses are responsible for seasonal epidemics in humans. IAV can be further classified by their primary host species including human influenza virus (hIAV), avian influenza virus (AIV), bat influenza virus, canine influenza virus (CIV), and swine influenza virus (swIAV) [97]. Based on the antigenic variations of the influenza hemagglutinin (HA) and neuraminidase (NA) surface glycoproteins, IAVs can be classified into 18 distinct HA (H1-H18) and 11 NA (N1-N11) subtypes [98]. hIAVs predominantly circulate as H1, H2, H3 and N1, N2 subtypes whilst AIVs harbor all H1 to H16 and N1 to N9 subtypes. H17N10 and H18N11 have been exclusively identified as bat influenza viruses. Since the 1900s, IAVs have caused five major pandemics, starting with the catastrophic 1918 Spanish Flu (H1N1), 1957 Asian flu (H2N2), 1968 Hong Kong flu (H3N2), 1977 Russian flu (H1N1), and the 2009 Swine flu (H1N1) [99]. Recently, in early 2025, Japan experienced a severe H1N1 influenza outbreak since 1999, which led to approximately 317,812 infections, possibly due to the lack of herd immunity to influenza since the COVID-19 pandemic [100]. Transmission of influenza viruses primarily occurs among humans through respiratory droplets from sneezes or coughs. Zoonotic transmission of influenza viruses occurs when animal influenza viruses are transmitted to humans via direct contact with infected animals, such as the AIVs in contaminated live birds [101]. As such, wild aquatic birds have been identified to be the primary natural host reservoir for most influenza A subtypes.

**Figure 6:**
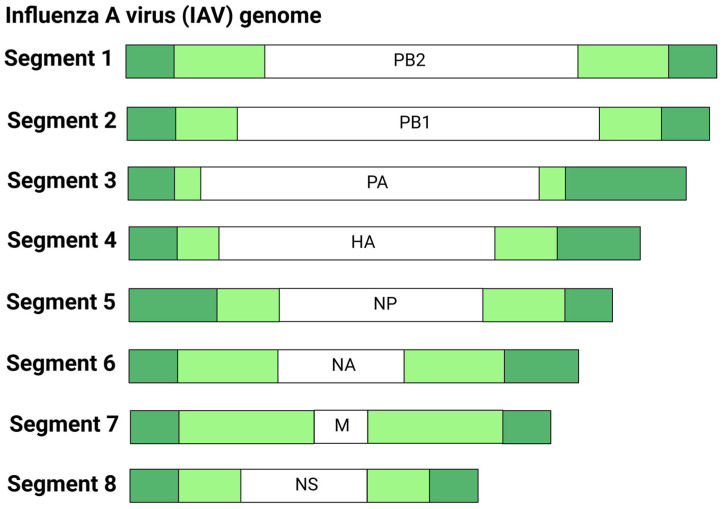
Schematic diagram of the Influenza A virus (IAV) genome structure. The IAV genome comprises eight negative-sense RNA segments encoding PB2, PB1, PA, HA, NP, NA, M, and NS (indicated in white boxes). The 3′ and 5′ untranslated regions (UTRs) (dark green) flank each segment, whilst the adjacent packing signals (light green) ensure proper virion assembly. The figure was created in https://BioRender.com.

Influenza viruses possess a segmented negative-sense single-stranded RNA genome that is organized into eight (A and B subtypes) or seven (C and D subtypes) RNA segments encoding 10 proteins [102]. Eight segments of the Influenza A single-stranded RNA are approximately 14 kb containing the PB2 (Segment 1), PB1 (Segment 2), PA (Segment 3), HA (Segment 4), NP (Segment 5), NA (Segment 6), M1 and M2 (Segment 7), NS1 and NS2/NEP (Segment 8) within the viral particle (Figure 6) [103]. The NS1 protein within Segment 8 has been suggested to impede interferon responses. Influenza viruses, particularly IAVs, pose a global health threat due to their ability to evolve, with their genome frequently undergoing mutations (antigenic drift) and reassortment of the eight RNA segments (antigenic shift), promoting immune evasion and the emergence of new pandemics. Antigenic drift occurs through the gradual accumulation of mutations in the HA or NA genes, resulting in changes in viral antigenicity that evade host immune recognition [104]. The segmented genomes of influenza A viruses facilitate antigenic shift, where an influenza A virus strain acquires new HA or NA genes via reassortment with an influenza virus of a completely different subtype [102]. Thus, antigenic shift might have led to the generation of influenza pandemic strains like the 1918 H1N1 Spanish flu. Based on recombinant studies of the 1918 Spanish flu virus, a unique combination of the influenza HA, NA, and PB1 genes was identified to contribute to its lethal pathogenicity [105]. Due to antigenic shift and drift, the formulation of influenza vaccines must be updated annually to effectively protect against circulating seasonal influenza strains [38]. With this, current influenza vaccines, which utilize traditional platforms such as inactivated and live-attenuated vaccines need to be reformulated to keep pace with these changes, as the process from designing to manufacturing normally would require approximately 6–8 months. The emergence of an outbreak caused by a novel influenza virus would necessitate rapid vaccine production, which could prove an impossible task utilizing the conventional vaccine platforms.

To overcome these underlying challenges, mRNA-based vaccine strategies against the influenza virus have made significant progress in development and have been rigorously studied in recent years. Petsch and colleagues (2012) were the first to utilize LNP-encapsulated mRNA encoding the HA from the H1N1 strain, which induced protective immunity [106]. A single dose of the mRNA-LNP vaccine candidate elicited robust neutralizing antibodies and T-cell responses and protected the immunized mice against a lethal viral challenge. This study established mRNA-LNP as a novel vaccine platform, which laid the foundation for the development of novel mRNA vaccines, including COVID-19 mRNA vaccines [107]. Magini et al. (2016) demonstrated that self-amplifying mRNA vaccine candidates encoding the H1N1 NP and M1 antigens delivered via LNP encapsulation elicited robust protective immunity in BALB/c mice [108]. These saRNA vaccine candidates induced both CD4^+^ Th1 and CD8^+^ T-cell responses and protected the mice against lethal challenge from both homologous (H1N1) and heterosubtypic (H3N2) influenza strains, highlighting the potential of the saRNA platform towards the development of broad-spectrum universal influenza vaccines. Vogel and colleagues (2018) compared two RNA vaccine platforms, non-amplifying RNA or saRNA, in terms of their immunogenicity and efficacy against influenza [109]. Immunization of BALB/c mice with either of the RNA vaccine platforms conferred equivalent protective immunity, but the saRNA vaccine candidate only required a 64-fold lower dose when compared to the synthetic RNA. Further studies revealed that a trivalent saRNA vaccine candidate encoding HA antigens from multiple influenza A and B strains (A/H1N1, A/H3N2, and B) conferred protection from a lethal H1N1 challenge in mice, once again reiterating the potential of the saRNA platform for vaccine development against viral diseases like influenza. Immunization of mice, ferrets, and rabbits with a nucleoside-modified mRNA-LNP vaccine encoding the purified full-length HA mRNA successfully elicited potent antibody responses against the HA head and stalk domains [110]. The vaccine candidate generated stalk-specific antibodies that conferred protection against homologous (H1N1) and heterosubtypic (H5N1) influenza strains in BALB/c mice, and a single dose was deemed sufficient to confer protection in mice. Thus, this study highlighted how this mRNA-LNP vaccine candidate could provide broadly protective immunity against influenza viruses.

Freyn and colleagues (2020) devised a multivalent nucleoside-modified mRNA-LNP vaccine candidate containing a combination of conserved influenza virus antigens, such as the HA stalk, M2, NA, and NP [111]. The vaccine candidate elicited a robust humoral immune response by eliciting antigen-specific antibodies, and immunized mice were protected against lethal viral challenge strains (H1N1, H5N8, cH6/1N5). Thus, this mRNA vaccine candidate, which could deliver multiple influenza virus antigens and was shown to elicit broad and potent immune responses, warrants further development as a promising universal influenza vaccine candidate. Chivukula et al. (2021) utilized the mRNA therapeutic platform (MRT) to develop multivalent mRNA-LNP vaccine candidates containing unmodified mRNAs encoding full-length HA and NA antigens from several influenza strains which include both seasonal and pandemic influenza strains [112]. Mice immunized with monovalent mRNA-LNP vaccine candidates (HA mRNA-LNPs or NA mRNA-LNPs) elicited functional antibody responses and were protected against lethal challenge with the H1N1 influenza strain. Co-encapsulation of HA and NA bivalent (H3H1, H3N2, N1N2) and quadrivalent mRNA (H1 + H3 + N1 + N2) combinations demonstrated no antigenic interference, with immunogenicity comparable to that of the monovalent vaccines. With this, the versatility of the MRT could enable the incorporation of other antigens, such as the SARS-CoV-2 antigens, should potential vaccines require broader protection against the co-circulating respiratory viruses. Interestingly, Arevalo et al. (2022) designed a multivalent nucleoside-modified mRNA-LNP vaccine candidate encoding the HA antigens from all 20 currently known IAV and IBV subtypes [113]. In ferrets and mice, the multivalent vaccine candidate elicited high levels of cross-reactive and subtype-specific antibodies against both HA head and stalk, and conferred protection against challenge from matched and mismatched viral strains. The authors hypothesized that the vaccine candidate conferred protection in immunized animals against mismatched viral strains, possibly through non-neutralizing mechanisms like antibody-dependent cellular toxicity (ADCC), whilst protection against antigenically matched viral strains was mediated by neutralizing antibody.

Van de Ven et al. (2022) developed a T-cell inducing universal influenza mRNA-LNP vaccine candidate targeting three conserved influenza proteins (NP, M1, PB1) (mRNA-Flu) of the H1N1 influenza virus [114]. By assessing the vaccine in ferrets to mimic naïve and influenza-primed humans, the vaccine candidate demonstrated elicitation of robust and broad T-cell responses in the blood, respiratory tract, and bone marrow, with enhanced protection in immunized ferrets against the zoonotic H7N9 avian influenza strain by reducing disease severity in influenza-primed ferrets. The findings suggested that this mRNA-LNP vaccine candidate could elicit cross-protective T-cell immunity against severe influenza disease and death from emerging zoonotic influenza infections, offering a potential strategy for the development of a universal influenza vaccine. A quadrivalent nucleoside-modified mRNA-LNP vaccine candidate targeting HA antigens from four seasonal influenza virus strains (A/H1N1, A/H3N2, B/Victoria, B/Yamagata) induced robust antibody responses against each subtype [115]. A single dose of the vaccine candidate, at high or low doses, was sufficient to fully protect the vaccine- immunized mice against lethal H1N1 challenge with no observed morbidity, highlighting the protective efficacy and immunogenicity of this quadrivalent mRNA vaccine candidate as an animal model. Recently, Leonard et al. (2024) developed a novel unmodified mRNA-LNP vaccine candidate that expressed both HA and NA glycoproteins from a single ORF using an artificial furin cleavage site and 2A ribosome-skipping sequences [116]. The novel vaccine candidate successfully elicited robust immune responses and protected vaccinated mice from a lethal dose of H3N2 challenge. An octavalent vaccine candidate combining four mRNA-LNPs encoding 4 HA and 4 NA antigens from A/H1N1, A/H3N2, B/Victoria, and B/Yamagata demonstrated strong immunogenicity against all four influenza strains in vaccinated mice and completely protected against lethal challenge from three different virus strains (A/H1N1, A/H3N2, B/Yamagata). These findings highlighted the potential of the mRNA-LNP vaccine platform to revolutionize the efficacy of seasonal and universal influenza vaccines.

With the success of the COVID-19 mRNA vaccines, major vaccine companies like Moderna, Pfizer-BioNTech, and Sanofi-Translate Bio have been developing mRNA vaccines against influenza or a combination of influenza and COVID-19. In 2021, Sanofi-Translate Bio’s monovalent mRNA-LNP influenza vaccine candidate entered a phase I clinical trial to evaluate its safety and immunogenicity against seasonal influenza [117]. Moderna’s quadrivalent mRNA vaccine candidate (mRNA-1010) for seasonal influenza, currently in phase III trials (NCT05827978), has demonstrated favorable safety profiles and robust immunogenicity against IAVs [118]. A phase III study was conducted to further evaluate the safety and immunogenicity of mRNA-1010 in three sequential stages involving adults ≥ 18, 18–64 and ≥65 years. [119]. The study demonstrated that a single 50 µg dose of mRNA-1010 elicited superior hemagglutination inhibition (HAI) antibody responses against all four vaccine-matched strains (A/H1N1, A/H3N2, B/Victoria, B/Yamagata) when compared to a licensed seasonal influenza vaccine. Robust immunogenicity was observed across all age groups, particularly in a high-risk group containing older adults (≥65 years). These results highlighted mRNA-1010 as a promising seasonal influenza vaccine candidate, leveraging the benefits of mRNA technology. Two other Moderna influenza mRNA vaccine candidates, mRNA-1020 and mRNA-1030, were developed to target four WHO-recommended seasonal strains (A/H1N1, A/H3N2, B/Victoria, B/Yamagata) [120]. Moderna has begun dosing participants in a phase I/II clinical trial (NCT05333289) to evaluate the safety, reactogenicity, and immunogenicity of these two mRNA vaccine candidates. A combined modified RNA vaccine candidate developed by Pfizer-BioNTech was reported to elicit robust immunogenicity against both influenza and SARS-CoV-2 with no safety concerns in a phase III trial (NCT06178991) [121]. It was comparable to the company’s licensed COVID-19 mRNA vaccine. In a parallel phase II trial, Pfizer’s trivalent influenza modified mRNA vaccine candidate “tIRV” demonstrated strong immunogenicity in adults aged 18–64 years when administered as a standalone vaccine (NCT06436703). While influenza pandemics are likely to occur again in the future, the emergence of novel influenza virus strains is relatively challenging to predict. An influenza pandemic arises when a new influenza virus, like the 2009 H1N1 swine flu virus, gains efficient and sustainable human-to-human transmissibility in a human population with minimal pre-existing immunity against the new influenza virus. Additionally, since avian and swine influenza viruses often cross species barriers to infect humans, vaccinating the natural hosts with mRNA vaccines matching the emerging strain when it first emerges could help prevent outbreaks.

### 5.5. mRNA Vaccines for Rabies Virus (RABV)

**Figure 7:**
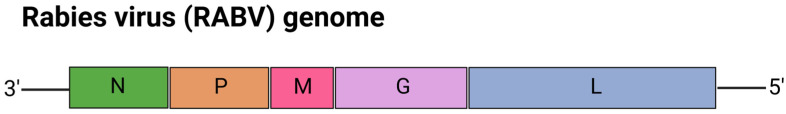
Schematic diagram of the rabies virus (RABV) genome structure. The RABV genome is a non-segmented negative-sense RNA genome encoding five structural proteins (N, P, M, G, L), flanked by 3′ and 5′ untranslated regions (UTRs). The figure was created in https://BioRender.com.

Rabies is an ancient neurotropic zoonotic disease caused by the rabies virus (RABV) belonging to the *Lyssavirus* genus within the *Rhabdoviridae* family [122]. Despite being preventable by vaccines, rabies still claims approximately 60,000–75,000 human lives annually, mostly in Asia and Africa, where access to prompt post-exposure prophylaxis (PEP) remains limited [123]. It is primarily transmitted through the saliva via bites or scratches of rabid dogs, which is responsible for 99% of the human rabies cases. Once a person is bitten or scratched by a rabid animal, they should immediately seek PEP care, which is thorough wound cleansing, prompt administration of a rabies vaccine, and the infiltration of rabies immunoglobulins (RIG) to avert the invariably fatal outcome. Once clinical symptoms like hydrophobia, aerophobia, hyperactivity, and hallucinations manifest, death is inevitable. RABV has an enveloped, bullet-shaped virion with a ~12 kb negative-sense, non-segmented, single-stranded RNA genome encoding five proteins: nucleoprotein (N), phosphoprotein (P), matrix protein (M), glycoprotein (G), and RNA-dependent RNA polymerase (L) (Figure 7) [124]. The RABV genome is composed of the L, N, and P proteins forming a stable ribonucleoprotein (RNP) complex that enables viral replication in the cytoplasm of the host cell. The sole surface viral protein, G, which mediates pathogenicity by facilitating receptor binding and membrane fusion, is also the major antigen for the induction of protective immunity [125]. Glycoprotein serves as the primary target for eliciting neutralizing antibodies against rabies. Egg-culture-based and cell-cultured-based technologies were utilized to culture live rabies viruses, which were inactivated by β-propiolactone to produce the inactivated rabies vaccine [126]. However, manufacturing of these inactivated vaccines is slow, and they are extremely laborious processes.

Advances in mRNA COVID-19 vaccine production have significantly accelerated the development of mRNA-based rabies vaccine candidates. Currently, several preclinical studies have demonstrated that mRNA rabies vaccine candidates could protect dogs, mice, monkeys, pigs, rabbits, and other animals against RABV infection [127]. In one of the earliest preclinical studies of RABV mRNA vaccine candidate, Schnee and colleagues (2016) assessed the immunogenicity and protective capacity of an optimized non-replicative mRNA vaccine candidate encoding the RABV glycoprotein (RABV-G) [128]. In mice, the vaccine candidate elicited robust CD4^+^ and CD8^+^ T-cell responses, with the CD4^+^ T responses being higher than those of a licensed inactivated vaccine (Rabipur or HDC). Immunized mice were also protected against lethal intracerebral challenge with the rabies CVS-11 strain. Apart from mice, a single dose of the RABV-G mRNA vaccine candidate demonstrated immunogenicity in domesticated adult pigs by inducing protective antibody responses, which exceeded the protective threshold. These findings highlighted the feasibility of the given RABV-G mRNA vaccine candidate in small (mice) and large animals like pigs. It is also cost-effective and has increased thermostability. Li and colleagues (2022) developed an optimized non-replicative mRNA vaccine (LVRNA001) encoding the RABV-G [129]. Two doses of the LNP-encapsulated LVRNA001 vaccine induced strong humoral and Th1-derived cellular immune responses and provided protective immunity against RABV in mice. Both immunized mice and dogs were protected, following an intracerebral challenge against a 50-fold lethal dose of the highly virulent RABV-BD06 strain. The results demonstrated the potential of LVRNA001 as an mRNA-LNP vaccine candidate for future prevention of rabies. However, as with all vaccine candidates, follow-up clinical trials are necessary to further evaluate the safety and efficacy of this mRNA vaccine in humans, given the limitations of this study in mice and dogs. Indeed, further studies demonstrated that two doses of the LVRNA001 vaccine elicited strong immune responses in pre- and post-exposure administration schedules, conferring 100% protection in dogs [130]. In cynomolgus macaques, LVRNA001 demonstrated no significant adverse effects on temperature, body weight, or biochemical markers. Toxicity studies of LVRNA001 in Sprague-Dawley rats also demonstrated no toxicity associated with the vaccine candidate. These findings showed that there were no significant vaccine-related adverse effects, highlighting LVRNA001 as a promising vaccine candidate for rabies prophylaxis and therapy.

Hellgren et al. (2023) investigated the immunogenicity of their unmodified mRNA-LNP vaccine candidate encoding the RABV-G and the traditional inactivated whole rabies virus vaccine (Rabipur) [131]. In rhesus macaques, the mRNA-LNP rabies vaccine candidate elicited higher levels of neutralizing antibodies, memory B-cells, plasma cells, and T-cells in comparison to Rabipur. Although both mRNA-LNP and Rabipur vaccines generated antibodies with similar somatic hypermutation, which indicated improved antibody binding affinity to the antigen, the mRNA-LNP platform provided an edge over Rabipur due to its broader and durable immune response and avoided the challenges associated with producing and purifying whole inactivated virus vaccines. Recently, Li et al. (2025) developed an mRNA-based vaccine candidate encoding the RABV-G encapsulated in a novel muscle-targeting LNP based on a proprietary STAR-002 formulation [132]. The RABV-G mRNA-LNP vaccine candidate elicited high virus- neutralizing antibody and IgG titers in a dose-dependent manner, outperforming the commercially available inactivated rabies vaccines. In mice, a single dose of the RABV mRNA-LNP was sufficient to confer 100% protection in pre-exposure challenge assays and 60% protection in post-exposure challenge, demonstrating its strong efficacy, safety, and cost-effectiveness (single dose) as an alternative vaccine to the traditional inactivated rabies vaccine.

While a considerable number of preclinical studies have explored the development of mRNA vaccines against rabies, only two vaccine candidates produced by CureVac (Germany), CV7201 and CV7202, have successfully advanced to phase I clinical trials to assess their immunogenicity, safety, and tolerability in humans [127]. CV7201 is a lyophilized, thermostable mRNA vaccine encoding the RABV-G in either free or complexed form with a cationic protein, protamine, whilst CV7202 is an upgraded formulation of CV7201, utilizing LNPs to deliver the mRNA. In 2017, 101 healthy participants were enrolled in a phase I clinical trial (NCT02241135), and they were administered with three doses of CV7201 intradermally or intramuscularly by either needle-syringe or needle-free devices [133]. Intriguingly, CV7201 was highly immunogenic and elicited enhanced neutralizing antibodies when injected with a needle-free device but not when injected with a needle-syringe. However, high doses of CV7201 of 80–640 µg were required to confer protection to participants. CV7201 was deemed to be relatively safe and reasonably well tolerated among the vaccinated participants in the clinical trial. To potentially reduce the dose, a phase I clinical study was conducted in Belgium and Germany (NCT03713086) to assess the immunogenicity, safety, and reactogenicity of different doses of the newly formulated CV7202 vaccine candidate in 55 healthy participants [134]. No serious adverse events were observed, but the 5 µg dose of CV7202 resulted in unacceptable reactogenicity in vaccinees. Two doses of the mRNA-LNP vaccine candidate (1 µg or 2 µg) were deemed sufficient to induce neutralizing antibody responses against rabies. Despite promising phase I results, transitioning these mRNA-based rabies vaccine candidates to phase II and III remains a significant challenge. As such, key uncertainties about the durability and efficacy of the immune responses elicited by the mRNA-LNP vaccines should be further evaluated in humans. Rabies disease remains a priority for global reduction of infections, with such efforts being shifted towards public awareness, mass dog vaccination globally, and improved PEP accessibility.

### 5.6. mRNA Vaccines for Zoonotic Arthropod-Borne Viruses

Rapid urbanization, international tourism, and unprecedented climate change have dramatically facilitated the spread of blood-sucking arthropods into new regions [135]. This has led to a significant rise in the emergence and re-emergence of arthropod-borne viruses (arboviruses) over the past few decades. Arboviruses represent a group of predominantly RNA viruses, whose members span multiple families such as *Flaviviridae* (dengue virus, Zika virus), *Phenuiviridae* (Rift Valley fever virus, Severe Fever with Thrombocytopenia Syndrome virus), *Rhabdoviridae* (vesicular stomatitis virus), *Bunyaviridae* (Crimean-Congo hemorrhagic fever virus), and *Togaviridae* (chikungunya virus) [136]. As such, these viruses rely on arthropod vectors (mosquitoes, ticks) for transmission by circulating in natural cycles involving livestock and wildlife, with humans often serving as incidental hosts. A number of vaccine candidates based on a wide range of platforms, including traditional vaccines, DNA vaccines, recombinant subunit-based vaccines, and adenovirus vector-based vaccines, have been developed, and indications of promising efficacy against respective virus infections have been obtained. The rapid success of mRNA vaccines during the COVID-19 pandemic has greatly accelerated mRNA vaccine development against these emerging and re-emerging arboviruses, such as dengue virus, Zika virus, and Rift Valley fever virus.

#### 5.6.1. Zika Virus (ZIKV)

**Figure 8:**
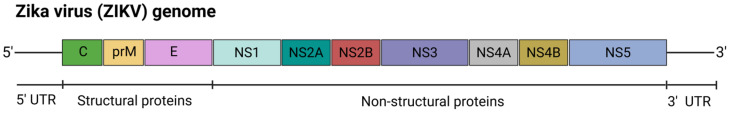
Schematic diagram of Zika virus (ZIKV) genome structure. ZIKV possesses a positive-sense RNA genome encoding a single polyprotein, which is cleaved into structural proteins (C, prM, E) and non-structural proteins (NS1, NS2A, NS2B, NS3, NS4A, NS4B, NS5). The figure was created in https://BioRender.com.

Richner et al. (2017) reported the development of a nucleoside-modified mRNA-LNP vaccine candidate encoding the Zika virus (ZIKV) prM and E structural proteins (Figure 8), which elicited high neutralizing antibody titers (~1/100,000) in three murine models [137]. ZIKA mRNA vaccine candidate protected immunocompromised AG129, immunocompetent BALB/c, and C57BL/6 mice against lethal ZIKV challenges, highlighting the potential of mRNA vaccines as a flexible platform against ZIKV. Two ZIKV mRNA-LNP vaccine candidates, mRNA-1325 and mRNA-1893, produced by Moderna have successfully advanced to phase I clinical trials to evaluate their immunogenicity and safety in healthy adult participants [138]. The first-generation ZIKV vaccine, mRNA-1325, encodes the prM-E proteins from an older Micronesia 2007 ZIKV strain, whilst mRNA-1893 encodes the prM-E proteins of a contemporary ZIKV strain (RIO-U1) [139]. The mRNA-1325 vaccine candidate was well tolerated among the vaccinees but elicited poor ZIKV-specific neutralizing antibodies (NCT03014089) [138]. On the other hand, the mRNA-1893 vaccine candidate was well tolerated and elicited robust neutralizing antibodies after two doses, irrespective of prior flavivirus exposure (NCT04064905), supporting its further development as the superior ZIKV vaccine candidate into the next stage of clinical trials. There is currently an ongoing phase II clinical trial of a two-dose mRNA-1893 vaccine candidate to assess its safety, tolerability, and reactogenicity in healthy individuals who are either flavivirus-seronegative or flavivirus-seropositive (NCT04917861).

#### 5.6.2. Dengue Virus (DENV)

**Figure 9:**
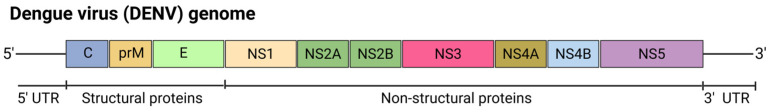
Schematic diagram of dengue virus (DENV) genome structure. DENV possesses a positive-sense single-stranded RNA genome encoding a single polyprotein which is cleaved into structural proteins (C, prM, E) and non-structural proteins (NS1, NS2A, NS2B, NS3, NS4A, NS4B, NS5). The figure was created in https://BioRender.com.

Given that Dengue virus (DENV) comprises four genetically related but antigenically distinct serotypes, the development of a dengue vaccine that can provide equitable protection against all four DENV serotypes remains a significant challenge (Figure 9) [140]. Achieving an ideal tetravalent protection against dengue is rather challenging, as immune responses often exhibit immunodominance of one or two serotypes over the others, leaving the vaccinated individual highly vulnerable to severe dengue upon subsequent infection with a heterologous DENV serotype. Hence, this imbalance can result in the induction of antibody-dependent enhancement (ADE), where sub-neutralizing or non-neutralizing antibodies can facilitate the enhancement of viral replication and spread, which can potentially lead to an increased risk of severe dengue infections [141]. The live-attenuated vaccine CYD-TDV, (Dengvaxia^®^) faces the challenge to overcome ADE and the need to elicit balanced protection against all four DENV serotypes. Several mRNA vaccine candidates against dengue have been demonstrated to be promising and are currently in the pre-clinical stage of development. In 2020, Zhang et al. (2020) developed three nucleotide-modified mRNA-LNP vaccine candidates (prME-mRNA; E80-mRNA; NS1-mRNA) against DENV-2 [142]. Each of the DENV mRNA-LNP vaccine candidates demonstrated robust immunogenicity by inducing DENV-2-specific IgG, eliciting strong neutralizing antibodies and antigen-specific T-cell responses. Either the E80 mRNA-LNP alone or the combined E80-NS1-mRNA-LNP vaccine was shown to provide complete protection in immunized mice challenged with DENV-2 in comparison to the NS1-mRNA-LNP vaccine, which only conferred partial protection.

Wollner et al. (2021) demonstrated the development of an mRNA-LNP vaccine candidate encoding both DENV-1 prM and E proteins [143]. Following a three-dose vaccination regimen, the DENV mRNA-LNP vaccine candidate induced cellular and humoral immunity with neutralizing antibody titers that were sufficient for protection against DENV-1. AG129 mice immunized with the prM/E mRNA-LNP vaccine candidate showed no morbidity and mortality after being challenged with a lethal DENV-1 strain. Intriguingly, this vaccine candidate elicited serotype-specific antibody responses, reducing the risk of developing ADE upon challenge with a live DENV-1 Western Pacific strain, highlighting the potential of the mRNA-LNP platform for dengue vaccines. Recently, He and colleagues (2022) designed two mRNA vaccine constructs (DENV-a and DENV-b) as a multi-target DENV mRNA-LNP vaccine candidate against DENV multiple serotypes [144]. This multi-target DENV mRNA-LNP vaccine candidate (DENV-ab) elicited high levels of neutralizing antibody titers against all DENV serotypes with minimal ADE. DENV-ab induced elevated cytokine productions such as IL-4, TNF-α, and IFN-γ, indicating a Th-1 biased response rather than Th-2. While these results are very promising, further validation and optimization of this DENV-ab in immunocompromised murine models like AG129 are important to further assess its efficacy and immunogenicity before advancing to clinical development.

#### 5.6.3. Rift Valley Fever Virus (RVFV)

**Figure 10:**
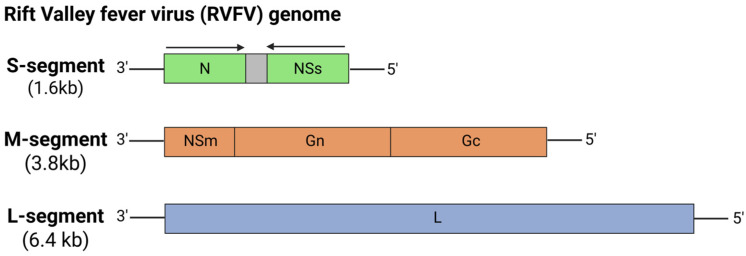
Schematic diagram of the Rift Valley fever virus (RVFV) genome structure. RVFV possesses a single-stranded, tripartite RNA genome consisting of three negative-sense segments. S-segment is ambisense, which encodes the nucleocapsid protein (N), is in the negative-sense orientation whilst the non-structural protein NSs, is in the positive-sense orientation. M segment encodes the NSm and the envelope glycoproteins, Gn and Gc. L-segment encodes the L-protein (RNA-dependent RNA polymerase). The figure was created in https://BioRender.com.

Rift Valley fever virus (RVFV) is a zoonotic arbovirus transmitted by mosquitoes that severely impacts livestock, causing fetal malformations, abortion storms, and high mortality in ruminant livestock (Figure 10) [145]. In humans, Rift Valley fever (RVF) can lead to encephalitis, hemorrhagic fever, and retinitis. First identified in Kenya’s Rift Valley in 1930, RVFV has since caused outbreaks across Africa and several countries in the Arabian Peninsula, raising concerns about its significant global threat due to vector expansion and unprecedented climate change [146]. Bian et al. (2023) developed six nucleotide-modified mRNA-LNP vaccine candidates encoding different regions of the RVFV Gn and Gc glycoproteins to address the lack of efficacy in licensed human vaccines for RVFV [147]. Out of the six RVFV vaccine candidates, the mRNA-LNP vaccine candidate encoding the full-length Gn and Gc glycoproteins (mRNA-LNP-GnGc) elicited both humoral and cellular immune responses, including high levels of neutralizing antibodies in BALB/c mice. Protection was observed when BALB/c mice were intraperitoneally challenged with a virulent RVFV rMP12 strain. The mRNA-LNP-GnGc vaccine candidate also induced strong neutralizing antibodies, T-cell responses, and humoral antigen-specific memory B cells, highlighting the mRNA-GnGc as a promising RVFV mRNA vaccine candidate with potent immunogenicity, although further studies are needed to determine the duration of the vaccine-induced immunity. Recently, Kitandwe et al. (2024) developed two LNP-encapsulated Venezuelan equine encephalitis virus (VEEV) self-amplifying RNA (saRNA) vaccine candidates encoding either the wild type RVFV Gn and Gc or modified (furin-T2A) RVFV Gn and Gc glycoproteins [148]. In BALB/c mice, both vaccine candidates elicited high levels of RVFV Gn-specific IgG antibodies in a dose-dependent manner. Interestingly, the modified furin-T2A variant, despite enhanced in vitro expression, demonstrated a significant reduction in neutralizing antibody responses, suggesting that conformational changes might have impaired the production of effective pseudovirus-neutralizing antibodies. While the results were highly promising, the study highlighted the wild type saRNA vaccine candidate as a promising vaccine candidate, and further studies are needed to comprehensively assess its immunogenicity and efficacy.

#### 5.6.4. Powassan Virus (POWV)

**Figure 11:**
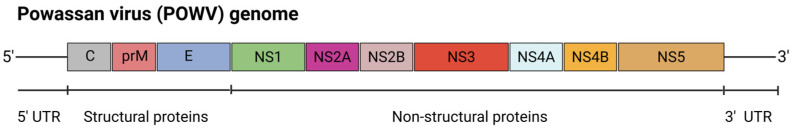
Schematic diagram of the Powassan virus (POWV) genome structure. POWV possesses a single-stranded positive-sense RNA genome encoding a single polyprotein, which is cleaved into structural proteins (C, prM, E) and non-structural proteins (NS1, NS2A, NS2B, NS3, NS4A, NS4B, NS5), flanked by 5′ and 3′ untranslated regions (UTRs). The figure was created in https://BioRender.com.

First discovered in 1958, the Powassan virus (POWV) is a tick-borne flavivirus isolated from a fatal case of encephalitis in a child from Powassan, Ontario (Figure 11) [149]. Human infections occur through spillover transmission from natural transmission cycles. Approximately 10% of severe neuroinvasive infections are fatal, with around half of the survivors suffering from long-term neurological complications [150]. Although relatively rare, the number of people falling ill from POWV infections had steadily increased in recent years [151]. Human cases of POWV have been reported in Canada, the United States, and Russia. There has been some encouraging progress in developing mRNA vaccines against POWV. VanBlargan et al. (2018) developed a nucleoside- modified mRNA-LNP vaccine candidate encoding the POWV prM and E genes, which elicited high titers of neutralizing antibody responses and provided protection against lethal challenges from POWV strains from lineage I (*Ixodes cookei* ticks) and lineage II (*Ixodes scapularis* deer ticks) [152]. Additionally, the vaccine also elicited cross-neutralizing antibodies against other tick-borne flaviviruses (TBFV) and protected the immunized mice against lethal challenge with the distantly related Langat virus, suggesting that this vaccine had uncharacterized inhibitory activity against multiple TBFV. Although it is in the early stages of development, the cross-protective potential and versatility of this mRNA-LNP vaccine candidate against multiple TBFV was demonstrated and might reduce the need for multiple vaccines in areas where these flaviviruses co-circulate.

#### 5.6.5. Crimean-Congo Hemorrhagic Fever (CCHF) Virus

**Figure 12:**
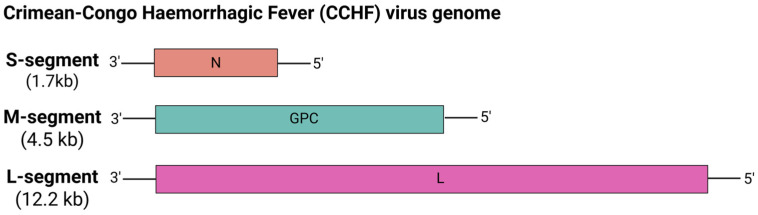
Schematic diagram of the Crimean-Congo Hemorrhagic Fever (CCHF) virus genome structure. CCHF virus possesses a tripartite negative-sense genome consisting of the S-segment, M-segment, and L-segment. The S-segment encodes the N protein. The M-segment encodes a precursor to the viral glycoproteins (GPC), which is further processed into surface glycoproteins Gn and Gc. L-segment encodes the L-protein (RNA-dependent RNA polymerase). The figure was created in https://BioRender.com.

Crimean-Congo Hemorrhagic Fever (CCHF) is a tick-borne viral disease caused by the CCHF virus that was first identified in the Crimean region in 1944 (Figure 12) [153]. The CCHF virus was later recognized as the same virus causing hemorrhagic disease outbreaks in the Congo basin, leading to its current name [154]. It is noted that the CCHF disease has a fatality rate of 10–40% [155]. CCHF transmission occurs via *Hyalomma* genus tick bites or by direct exposure to the blood/tissues of infected animals, especially during slaughtering. Clinical signs of CCHF include severe hemorrhagic fever, such as bleeding in the gums, blood detected in the urine, sputum, vomit, and even intra-abdominal hemorrhage [156]. CCHF is regarded as one of the most widespread arboviral diseases globally, spanning regions from southern Russia and the Black Sea to the southern tip of Africa [154]. In recent decades, CCHF has slowly expanded into new regions such as Albania, Turkey, and Georgia between 2001 and 2009. Regions like south-western Russia and Central Africa have reported human cases of CCHF infection after prolonged periods of absence, undermining its status as a global emerging threat. Recent breakthroughs in vaccine development have expanded opportunities for preventing CCHF.

Utilization of the mRNA vaccine platform represents a novel approach that could work complementarily with current existing vaccine platforms, such as the promising CCHF adenovirus-vectored vaccine candidate, CAdOx2-CCHF, developed by the Oxford Vaccine Group, which is currently undergoing first-in-human clinical trials [157]. Appelberg et al. (2022) assessed the efficacy of two nucleoside-modified mRNA-LNP vaccine candidates encoding either the CCHFV glycoproteins (Gc and Gn) or the nucleoprotein (N) [158]. IFNAR^-/-^ mice immunized with either CCHF vaccine candidates survived with no clinical manifestations following challenge with a lethal dose of the CCHFV Nigerian strain, despite lacking sterilizing immunity. Both vaccines elicited strong humoral and cell-mediated immune responses, with both Gc/Gn and N induced T cells against CCHFV. In another study, Leventhal and colleagues (2024) demonstrated that an alphavirus-based self-replicating RNA vaccine candidate expressing the CCHFV NP and CCHFV Gc proteins (repNP + repGc) could elicit non-neutralizing anti- nucleoprotein (NP) antibodies and Gc-specific T-cell responses [159]. To evaluate the durability and efficacy of the vaccine candidate, immunized mice when challenged with a lethal CCHFV strain were protected for at least one year post-vaccination, with 100% survival up to 9 months and 80% survival within 12 months post-vaccination. These findings highlighted the potential of this CCHFV vaccine to provide durable immunity, supporting its further development in clinical trials. Follow-up studies by Hawman et al. (2024) further evaluated the CCHFV repNP/repGc vaccine candidate in a rhesus macaque (*Macca mulatta*) CCHF model [160]. Prime-boost vaccination of the rhesus macaques with the repNP/repGc elicited robust non-neutralizing humoral and cellular immune responses, and conferred protection against CCHFV challenge with the passaged-CCHFV strain Hoti. This study further supported the development of this vaccine candidate and established rhesus macaques as an alternative non-human primate model apart from cynomolgus macaques (*Macca fascicularis*) in CCHF vaccine research. Chen et al. (2024) designed three nucleoside-modified mRNA-LNP vaccine candidates, which encode glycoproteins Gn (vLMn), Gc (vLMc), and GnNSmGc (vLMs) which were delivered via microfluidics [161]. The vLMc vaccine candidate elicited stronger B-cell and T-cell immune responses when compared to vLMn, whilst vLMs intriguingly induced weaker immune responses, suggesting that the NSm linker might impair vLMs immunogenicity. This study supported the utilization of Gc as a dominant antigen, supplemented by Gn, and the avoidance of Nsm in the future design of the CCHFV vaccine candidate against CCHF. The development of vaccines against CCHF would help to mitigate transmission from animal reservoirs such as ticks to humans, while also reducing CCHF disease burden on public health systems [162].

#### 5.6.6. Severe Fever with Thrombocytopenia Syndrome (SFTS) Virus

**Figure 13:**
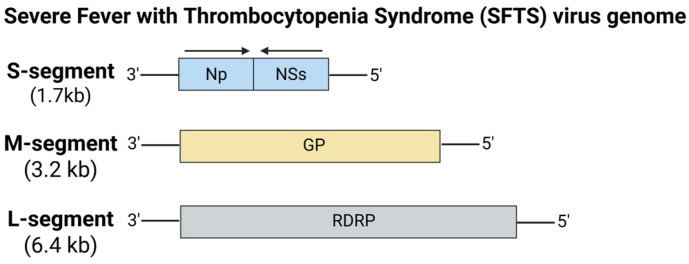
Schematic diagram of the Severe Fever with Thrombocytopenia Syndrome (SFTS) virus genome structure. SFTS virus possesses a tripartite negative sense genome consisting of the S-segment, M-segment, and L-segment. The S-segment is ambisense, which encodes the nucleocapsid protein (Np) in the negative-sense orientation, and the non-structural protein NSs is in the positive-sense orientation. M-segment encodes a precursor to the viral glycoprotein GP, which is further processed into surface glycoproteins Gn and Gc. L-segment encodes the L-protein (RNA-dependent RNA polymerase). The figure was created in https://BioRender.com.

Severe Fever with Thrombocytopenia Syndrome virus (SFTSV) is an emerging tick-borne virus known to induce severe fever, leukocytopenia, and thrombocytopenia, often associated with high mortality rates of up to 30% in humans (Figure 13) [163]. Being a member of the *Bandavirus* genus of the *Phenuiviridae* family, SFTSV was first isolated in Hubei Province, China, in 2009 [164]. Later, its presence was confirmed across East Asian countries in Japan, Taiwan, South Korea, and it even made its way to Vietnam and Thailand, suggesting its potential in spreading from endemic SFTSV infection areas [165,166,167]. SFTSV is primarily transmitted through the bites of an infected Asian longhorned tick, *Haemaphysalis longicornis*, though secondary routes such as nosocomial transmission from infected individuals to healthcare workers and sporadic animal to human transmission have been widely observed [168,169]. Climate change has facilitated the expansion of the habitat for the *Haemaphysalis* tick into new geographical regions such as North America, New Zealand, and Australia, increasing the risk of introducing SFTSV to other tick species [170]. Recent reports of *Haemaphysalis* ticks spreading across the United States correspond with the growing concerns that SFTSV could potentially emerge as a significant public health threat in affected areas [171].

Since no licensed vaccines are currently available for SFTSV, there are ongoing efforts to develop vaccines on various vaccine platforms, including mRNA vaccines against SFTS. Kim et al. (2023) developed mRNA vaccine candidates targeting the head of the soluble Gn glycoprotein (sGn-H) of the SFTSV [172]. Two mRNA-LNP vaccine candidates, one encoding the sGn-H and another containing the sGn-H fused with self-assembling 24-mer ferritin (FT) nanoparticles (sGn-H-FT), were developed to test their immunogenicity in mice. In immunocompromised AG129 mice, both SFTSV vaccine candidates elicited strong humoral immunity with neutralizing antibodies lasting up to 12 weeks following booster. Mice immunized with each vaccine candidate were fully protected from lethal challenge with the SFTSV HB29 strain, despite initially exhibiting significant weight loss. These data demonstrated the potential of these mRNA-LNP vaccine candidates, conferring protective immunity against SFTSV infection and associated disease pathogenesis. Another research group developed an mRNA-LNP vaccine expressing the SFTSV Gn glycoprotein, which demonstrated elicitation of robust neutralizing antibodies and Gn-specific T-cell responses [173]. Immunocompetent C57BL/6 mice vaccinated with the SFTSV mRNA-LNP vaccine were completely protected from lethal SFTSV infection, with minimal weight loss and reduced liver and spleen damage being observed. As individuals aged above 50 years are most susceptible to SFTS, further research is needed to assess the efficacy of these vaccine candidates in older animal models. Recently, Lu et al. (2024) developed a nucleoside-modified mRNA-LNP vaccine candidate encoding the full-length glycoprotein GP (Gn + Gc), which successfully induced robust humoral and Th-1 biased cell-mediated immune responses in immunocompetent BALB/c mice [174]. Both low and high doses of the GP vaccine candidate also completely protected immunized mice from lethal SFTSV challenge and provided durable immunity for at least five months. Intriguingly, the GP mRNA-LNP vaccine candidate also conferred cross-protection against other *bandaviruses* like Heartland virus and Guertu virus, despite the absence of cross-neutralizing antibodies, indicating that cell-mediated immune response might be responsible for broad-spectrum protection. These findings demonstrated the potential of the GP mRNA-LNP vaccine candidate against SFTSV and other *bandaviruses*, offering a promising strategy for developing broad-spectrum vaccines. With SFTSV potentially spreading beyond the endemic regions of East Asia, there is an urgent need for increased research efforts to develop safe and effective vaccines against SFTSV.

### 5.7. mRNA Vaccines for Lassa Virus (LASV)

**Figure 14:**
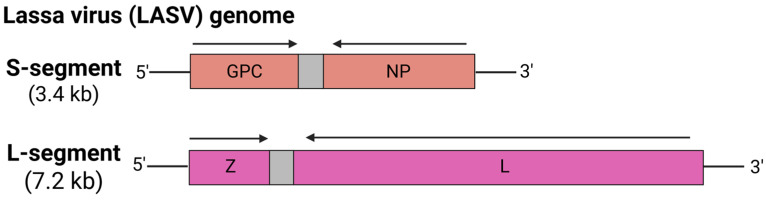
Schematic diagram of the Lassa virus (LASV) genome structure. LASV genome comprises two ambisense RNA segments. The S-segment contains the glycoprotein precursor (GPC) in positive-sense orientation, whilst the nucleoprotein (NP) is in the negative-sense orientation. The L-segment contains the Z matrix protein, which is in the positive-sense orientation, whilst the L-protein (RNA-dependent RNA polymerase) is in the negative-sense orientation. The figure was created in https://BioRender.com.

Lassa fever is an acute viral zoonotic disease caused by the Lassa virus (LASV) belonging to the *Mammarenavirus* of the *Arenaviridae* family (Figure 14) [175]. First identified in 1969 in Lassa, Nigeria, LASV is maintained by its natural host reservoir, which was identified to be the multimammate rat, *Mastomys natalensis*. It sheds the virus in its urine and feces without manifesting clinical symptoms. LASV infection in humans primarily occurs through exposure to household or food items contaminated with urine and feces from infected *Mastomys* rats, or possibly via direct contact with the rodents themselves. Although 80% of infections are asymptomatic, the case fatality rate of 1% could rise to 18% in hospitalized patients and up to 25% during outbreaks. LASV is endemic in West Africa, where it is responsible for approximately 300,000 infections and 5000 deaths annually, which is the second largest global health burden after DENV [176]. Lassa fever is highly prevalent across West Africa, most particularly in Guinea, Liberia, Sierra Leone, and Nigeria, where *Mastomys* rats are most ubiquitous in residential areas [177]. Over the last several years, Nigeria has experienced significant LASV outbreaks, a trend persisting in 2024, with 928 cases and 162 deaths being reported [178]. The accurate number of LASV infections remains challenging due to the asymptomatic nature of the infections, underreporting, and limitations in proper diagnosis. LASV-associated seroprevalence studies suggested that the incidence of LASV superseded the number of reported Lassa fever cases [179]. Due to the lack of available LASV vaccines, LASV remains a priority pathogen for global health surveillance and intervention efforts.

With the success of the COVID-19 mRNA vaccines, several LASV mRNA vaccine candidates are currently in development and are being evaluated in preclinical studies. Ronk and colleagues (2023) reported on the efficacy of two nucleoside-modified LASV mRNA-LNP vaccines encoding either the wild type or prefusion-stabilized glycoprotein complex (GPC) of the LASV strain Josiah [180]. In outbred Hartley guinea pigs, both vaccine candidates induced robust binding antibody responses, specifically to the prefusion-stabilized GPC, and interestingly, neutralizing antibodies were readily detected in several of the vaccinated guinea pigs. All animals vaccinated with either one of the LASV vaccine candidates survived the challenge with a lethal dose of the LASV strain Josiah without severe disease manifestations, suggesting that protection might be associated with strong Fc-mediated effects. This study highlighted the immunogenicity and efficacy of these mRNA-LNP vaccine candidates against LASV, though further studies are needed to elucidate the mechanisms and their durability. Hashizume et al. (2024) evaluated two nucleoside-modified LASV mRNA-LNP vaccines expressing the LASV glycoprotein precursor (LASgpc) or lymphocytic choriomeningitis virus nucleoprotein (LCMnp) as a safer alternative to the handling of live LASV in C57BL/6 mice [181]. Immunization with LASgpc or LCMnp in mice conferred protection from lethal challenge with the recombinant lymphocytic choriomeningitis virus (LCMV) expressing LASV GPC, despite negligible neutralizing activity in the plasma of LASgpc-immunized mice. Two doses of the LASV mRNA-LNP vaccine candidates induced strong humoral and cell-mediated immune responses, and the protective effect has been suggested to correlate with anti-LASV CD8^+^ T-cell responses. Further validation of these LASV vaccine candidates in authentic challenge models, potentially involving nonhuman primates with live LASV is needed. In recent years, mRNA vaccines have demonstrated their safety, scalability, and production speed, making them highly viable candidates for combating diseases like LASV [178]. However, deploying these LASV mRNA-based vaccines in low-income regions in West Africa could pose some challenges due to their inherent stability, cold-chain storage requirements, and the need for trained personnel for vaccine administration. An overview of mRNA vaccine development for zoonotic viral diseases is presented in Table 1.

## 6. Future Directions in Preventing Infections Caused by Emerging and Re-Emerging Pathogens

The complex nature of factors driving zoonotic diseases highlights the critical role of One Health in safeguarding animal, human, and environmental health. Initially introduced as “One Medicine” by Rudolf Virchow in the 19th century, One Health is an integrated and collaborative approach that recognizes the interconnectedness of animals, humans, and the environment [183,184]. Implementing a One Health strategy is crucial for minimizing the burden and enhancing the prevention and control of zoonoses [185]. One of the critical steps towards this implementation is assessing and compiling available One Health approaches, policies, and strategic frameworks. Predicting, detecting, preventing, and responding to potential global health threats like the recent COVID-19 pandemic is of utmost priority [184]. Notably, 70–80% of emerging and re-emerging infectious diseases originate from animals, underscoring the persistent risk of zoonotic spillover.

The One Health framework integrates the public health, veterinary, and environmental sectors. This collaborative approach is important for controlling zoonotic diseases such as rabies, Rift Valley Fever, and Lassa fever. The One Health policy aims to reduce pollution and maintain our food, water, and nutrition security. While developing vaccines provides a proven and effective means of preventing zoonotic diseases, they are not fully utilized to their potential. As such, massive opportunities for partnerships between health sectors to implement intervention strategies and vaccine development are often overlooked [186]. To overcome this, it would be of great interest if information or progress of vaccine research and development were shared and streamlined between the human and animal sectors. This can be potentially accomplished through initiatives such as joint training programs, interprofessional conferences, and even vaccine development progress meetings, which can highlight how advances in the vaccine of interest can benefit multiple species. By fostering such collaborations, stakeholders or authorities can evaluate the efficacy of vaccines of interest across the One Health spectrum and expedite the progress should there be a need for it. Given the global impact of emerging zoonotic diseases, developed nations should invest in building and reinforcing surveillance systems in resource-limited countries [187]. International health regulations should emphasize developing responsive capabilities, raising awareness, and promoting interdisciplinary collaborations and coordination, to prepare for a potential zoonotic global threat.

Following the Zika virus outbreak in 2015, a panel of experts across multiple disciplines gathered in Geneva to develop a framework for prioritization of pathogens under the WHO Blueprint for R&D preparedness [188]. The WHO initiative focused on severe emerging diseases that could potentially trigger public health emergencies, mainly those with inadequate treatments and preventive measures. Most importantly, the multidisciplinary experts identified seven priority diseases requiring urgent R&D: Crimean-Congo hemorrhagic fever (CCHF), highly pathogenic emerging coronaviruses of relevance to humans, filovirus diseases (e.g., Ebola, Marburg), Lassa fever, Nipah, Rift Valley fever, and lastly, R&D preparedness for a new disease. Three additional diseases were also prioritized due to their severity, which require urgency for intervention: chikungunya, Zika, and severe fever with thrombocytopenia syndrome (SFTS). The concept was to enhance global readiness by developing vaccines, diagnostics, and therapeutics, and addressing key scientific gaps. These efforts proved crucial years later, enabling a rapid response to COVID-19 by countries via accelerated vaccine development and the application of public health measures. In 2022, the WHO convened another meeting to review pandemic strategies that were utilized to mitigate COVID-19 and discuss preparedness for “Pathogen X” [189,190]. The One Health approach was also emphasized as essential for early prediction, detection, and mitigation of the next emerging pathogen.

Conventional vaccine platforms, such as live-attenuated and inactivated, often involve prolonged development timelines, posing a critical challenge during sudden outbreaks. Thus, one of the greatest impacts of mRNA vaccines lies in their ability to facilitate rapid responses to emerging zoonotic pandemics [191]. In the event of a future pandemic, mRNA vaccine technology offers distinct advantages as it is rapid, scalable, and cost-effective for production in a cell-free system. Once the genetic sequence of a pathogen has been characterized and the protective antigen(s) have been found, researchers can swiftly generate an mRNA construct encoding its key antigenic targets. Unlike traditional vaccines, mRNA platforms eliminate the need for hazardous and labor-intensive live virus cultivation, making vaccine development both faster and safer. This modular design suggests that mRNA vaccines could be efficiently adapted for rapidly emerging viruses [192]. mRNA vaccines remain promising in combating zoonotic pathogens, causing novel outbreaks [191]. The rapid adaptability of mRNA technology could allow for preemptive vaccine development against zoonotic pathogens detected in animal reservoirs, thereby curbing spillover risks. When coupled with established global surveillance systems, mRNA vaccine technology could become pivotal in helping to halt localized outbreaks before they escalate into a widespread emerging pandemic [193].

## 7. Conclusions

The integration of One Health approaches with the mRNA vaccine technology marks a transformative era in global health, offering a unified approach to combat infectious diseases at the human–animal–environment interface. With decades of demonstrated success in both human and veterinary medicine, traditional vaccine platforms provide established safety profiles, cost-efficient production, and dependable thermostability. However, these traditional vaccines typically involve the development of a single or multiple strains, potential virulence reversion risk particularly with live-attenuated strains, and reduced efficacy against rapidly evolving viral pathogens. In contrast, mRNA vaccines with their rapid adaptability, accelerated development, and manufacturing time demonstrated remarkable success during the COVID-19 pandemic and now hold immense potential for addressing emerging zoonotic viral threats. Unlike conventional vaccines, these mRNA vaccines can overcome the limitations of conventional vaccine platforms that require lengthy periods of production involving complex processes. Yet mRNA vaccines face significant underlying challenges, including high costs associated with expensive materials (e.g., LNP) and cold-chain dependence due to RNA instability [194]. Researchers are addressing these limitations through innovations like lyophilized mRNA-LNP vaccine formulations (e.g., CV7201) to enable stable long-term storage at 2–8 °C [127,195], while exploring cost-effective delivery alternatives such as the use of polymer-based nanoparticles and cationic nanoemulsions [196,197]. These would enable mRNA vaccines to be more accessible and affordable globally. To maximize the benefits of mRNA vaccine technology, funding should prioritize long-term safety studies to address public vaccine hesitancy, scale-up technologies to reduce production costs in low-income countries, as well as improving mRNA vaccine scalability and thermostability. In conclusion, One Health approaches are essential for global health security, emphasizing prevention in an increasingly interconnected world. The mRNA vaccine technology will pave the way for a resilient and interconnected ecosystem that will safeguard both human and animal populations against emerging and re-emerging viral pathogens.

## Figures and Tables

**Table 1:** Overview of mRNA vaccine development for zoonotic viral diseases.

Vaccine Name	Target Virus	Target	Vaccine Design	Findings	Development Stage	Ref.
MERS-COV RBD-mRNA vaccine	MERS-CoV	RBD domain of S protein	Nucleoside-modified mRNA-LNP vaccine candidate	Elicited durable, potent, and broad cross-neutralization antibodies against multiple MERS-CoV strains Induced robust cellular immune responses Conferred complete protection against MERS-CoV challenge in mice	Pre-clinical BALB/c mice	[67]
BNT162b2 (Cominarty by Pfizer-BioNTech)	SARS-CoV-2	Full-length S protein of SARS-CoV-2	Nucleoside-modified mRNA-LNP containing the full-length S protein of SARS-CoV-2 with S2 subunit containing two proline substitutions (amino acid positions 986 and 987) in prefusion conformation	Elicited a robust immune response characterized by the production of high levels of SARS-CoV-2-specific neutralizing antibodies and strong T-cell responses Two doses conferred 95% protection against COVID-19 in individuals aged ≥ 16 years or older	Approved for use in December 2020	[72,73]
mRNA-1273 (Spikevax by Moderna)	SARS-CoV-2	Full-length S protein of SARS-CoV-2	Nucleoside-modified mRNA-LNP containing full-length S protein of SARS-CoV-2 with two proline substitutions within the S2 subunit in prefusion conformation and an intact furin cleavage site	Elicited robust and durable immune response against SARS-CoV-2 following vaccination with two doses of the mRNA-1273 A third dose significantly increases neutralizing antibody levels Demonstrated 94.1% efficacy against symptomatic COVID-19 following Day 64 post-vaccination follow-up	Approved for use in January 2021	[182]
Nucleoside- modified mRNA-LNP vaccine	EBOV	Ebola virus glycoprotein (EBOV-GP)	Two nucleoside-modified mRNA-LNP vaccine candidates, EBOV-GP with wild type EBOV-GP signal peptide (Vaccine A) and EBOV-GP with human Igκ signal peptide (Vaccine B)	Vaccine B elicited higher GP-specific IgG and neutralizing antibody titers when compared to Vaccine A Two doses of either vaccine conferred 100% protection in guinea pigs against a lethal guinea pig-adapted EBOV challenge	Pre-clinical Guinea pigs strain Harley	[80]
sHeVG mRNA-LNP vaccine	NiV	Soluble Hendra virus glycoprotein (sHeVG)	Nucleoside-modified mRNA-LNP vaccine candidate encoding the sHEVG	No detectable pre-challenge neutralizing antibodies in the plasma of immunized hamsters A single dose of the mRNA-LNP vaccine protected 70% of the immunized Syrian hamsters against the lethal NiV challenge Surviving hamsters displayed robust NiV-specific IgG and neutralizing antibodies post-challenge infection	Pre-clinical Syrian hamsters (*Mesocricetus auratus*)	[91]
Nucleoside- modified mRNA-LNP vaccine	NiV	NiV prefusion-stabilized fusion glycoprotein (pre-F+G)	Nucleoside-modified mRNA-LNP vaccine candidate encoding the pre-F+G	Elicited strong neutralizing antibodies and robust T-cell immunity by inducing T follicular helper (Tfh) and CD8^+^ T-cell responses	Pre-clinical CB6F1/J mice	[92]
NiV sG mRNA-LNP vaccine	NiV	NiV soluble glycoprotein (NiV sG) from a Malaysian NiV strain (NiV-M)	Nucleoside-modified mRNA-LNP vaccine candidate encoding the NiV-M sG	Elicited potent antigen-binding and virus-neutralizing antibodies following the second booster dose immunization Induced both CD4^+^ and CD8^+^ T-cell responses	Pre-clinical White-Landrace-Hampshire cross-bred pigs	[93]
mRNA NiV G-NP	NiV	60 head domains of NiV glycoproteins	mRNA nanoparticle (NP) vaccine displaying 60 head domains of NiV glycoprotein	Elicited a robust anti-NiV G humoral response and NiV neutralizing antibody response with high serum NiV neutralizing titers using a pseudotyped NiV system	Pre-clinical CB6F1/J mice	[94]
mRNA-1215 (Moderna)	NiV	Secreted pre-F/G of the NiV-M	mRNA vaccine encoding the secreted pre-F/G of the NiV-M	Currently being evaluated for its safety and immunogenicity in healthy adult participants aged 18 to 60 years	Phase I NCT05398796 (Completed)	[95]
mRNA-LNP vaccine	Influenza virus	Hemagglutinin (HA)	mRNA-LNP vaccine candidate encoding the HA from the H1N1 influenza strain	Vaccinated mice elicited robust neutralizing antibodies and T-cell responses Conferred complete protection against a lethal viral challenge	Pre-clinical BALB/c mice	[106]
SAM M1-NP mRNA-LNP vaccine	Influenza virus	Nucleoprotein (NP) and matrix protein 1 (M1)	mRNA-LNP vaccine candidate encoding theH1N1 NP and M1 antigens	Induced robust NP-specific CD8^+^ T-cells and polyfunctional CD4^+^ Th1 cells 78% survival against lethal homologous (H1N1) challenge, whilst achieving 100% survival against lethal heterosubtypic (H3N2) challenge	Pre-clinical BALB/c mice	[108]
sa-RNA vaccine/non-replicating synthetic RNA vaccine	Influenza virus	HA from a model influenza strain (monovalent) HA from multiple influenza A and B strains (A/H1N1, A/H3N2, B/Massachusetts) (trivalent)	Two RNA vaccine candidates, saRNA and non-replicating RNA	Both monovalent sa-RNA and non-replicating RNA vaccines conferred equivalent protective immunity, but sa-RNA required a 64-fold lower dose to achieve protective immunity when compared to the non-replicating RNA vaccine Trivalent saRNA vaccine protected the immunized mice against sequential H1N1 and H3N2 lethal challenges	Pre-clinical BALB/c mice	[109]
Nucleoside-modified mRNA-LNP vaccine	Influenza virus	Fast protein liquid chromatography (FPLC)-purified full-length HA protein	Nucleoside-modified mRNA-LNP vaccine candidate encoding the full-length HA from H1N1 (A/Cal09)	Elicited potent antibody responses against the HA head and stalk domains in mice, rabbits, and ferrets Protected immunized mice against homologous (H1N1) and heterosubtypic (H5N1) influenza strains	Pre-clinical BALB/c mice Rabbits Ferrets	[110]
Nucleoside-modified mRNA-LNP vaccine	Influenza virus	HA stalk, matrix-2 ion channel (M2), neuraminidase (NA) and nucleoprotein (NP)	Multivalent nucleoside- modified mRNA-LNP vaccine candidate encoding a combination of conserved influenza virus antigens from different H1N1 strains	Single dose elicited a robust humoral immune response by eliciting antigen-specific antibodies, with NA-specific antibodies displaying strong neutralization activity Immunized mice were protected from a diverse range of influenza A viruses (H1N1, H5N8, cH6/1N5)	Pre-clinical BALB/c mice	[111]
Unmodified mRNA-LNP vaccines	Influenza virus	Full-length HA and NA antigens	Monovalent/multivalent mRNA-LNP vaccine candidate encoding full-length HA or NA, or both, from several seasonal and pandemic influenza strains	Mice immunized with the monovalent vaccine candidate elicited robust functional antibody responses and conferred sufficient protection against lethal viral challenge	Pre-clinical BALB/c mice Cynomolgus macaques	[112]
Nucleoside-modified mRNA-LNP vaccine	Influenza virus	20 HA antigens	Nucleoside-modified mRNA-LNP vaccine candidate encoding 20 HA antigens from all known IAV and IBV subtypes	Elicited high levels of cross-reactive and subtype- specific antibodies in mice and ferrets Conferred protection in both mice and ferrets against matched and mismatched viral strains	Pre-clinical Mice Ferrets	[113]
Universal influenza mRNA-LNP vaccine (mRNA-Flu)	Influenza virus	NP, M1 and polymerase basic protein 1 (PB1) of the H1N1	Universal influenza mRNA-LNP vaccine candidate targeting conserved influenza proteins	Elicited robust and broad T-cell responses in blood, bone marrow and respiratory tract Vaccination with a booster dose enhanced protection against the zoonotic H7N9 avian influenza strain, especially in influenza-primed ferrets	Pre-clinical Ferrets	[114]
Quadrivalent nucleoside-modified mRNA-LNP vaccine	Influenza virus	HA antigens	Nucleoside-modified mRNA-LNP vaccine candidate targeting HA from four seasonal influenza strains (A/H1N1, A/H3N2, B/Victoria, B/Yamagata)	Elicited robust antibody responses against all four subtypes Provided complete protection against lethal H1N1 viral challenge	Pre-clinical C57BL/6 mice	[115]
Unmodified mRNA-LNP vaccine	Influenza virus	HA and NA antigens	Unmodified mRNA-LNP vaccine candidate expressing both HA and NA from a single ORF using an artificial furin cleavage site and 2A ribosome-skipping sequences	Induced high levels of functional neutralizing antibodies and protected mice from a lethal dose of H3N2 challenge Octavalent vaccine encoding 4 HA and 4 NA antigens (A/H1N1, A/H3N2, B/Victoria, B/Yamagata) demonstrated strong immunogenicity and completely protected mice against lethal challenge from three virus strains (A/H1N1, A/H3N2, B/Yamagata)	Pre-clinical Mice Ferrets	[116]
Monovalent mRNA-LNP influenza vaccine (Sanofi-Translate Bio)	Influenza virus	HA	Monovalent mRNA-LNP vaccine encoding the HAprotein of the A/H3N2 seasonal influenza strain	Not available	Phase I	[117]
mRNA-1010 (Moderna)	Influenza virus	HA	Quadrivalent mRNA vaccine expressing HA proteins from four seasonal influenza viruses (A/H1N1, A/H3N2, B/Victoria, B/Yamagata)	A single 50 µg dose of mRNA-1010 elicited superior HAI antibody responses in all four vaccine strains when compared to a licensed seasonal influenza vaccine Robust immunogenicity was observed across all age groups, especially in older adults (≥65 years)	Phase III NCT05827978 (Completed)	[118,119]
mRNA-1020 and mRNA-1030 (Moderna)	Influenza virus	HA and NA	mRNA vaccine encoding HA and NA glycoproteins of 8 influenza strains	Not available	Phase I/II NCT05333289 (completed)	[120]
Combined modified RNA vaccine (Pfizer-BioNTech)	Influenza virus and COVID-19	Not available	Not available	Elicited robust immunogenicity against both influenza and SARS-CoV-2 with no safety concerns	Phase III NCT06178991 (Completed)	[121]
Trivalent influenza vaccine (tIRV) (Pfizer-BioNTech)	Influenza virus	Not available	Not available	Demonstrated strong immunogenicity in adults aged 18–64 years Elicited robust influenza A responses when compared to licensed vaccine Showed lower geometric mean titers (GMT) and seroconversion against the influenza B strain	Phase II NCT06436703 (Completed)	[121]
RABV mRNA vaccine	RABV	RABV glycoprotein (RABV-G)	Non-replicating mRNA vaccine encoding the RABV-G	Elicited high levels of neutralizing antibodies and antigen-specific CD4^+^ and CD8^+^ T-cell responses in mice Protected immunized mice against a lethal intracerebral challenge infection Induced protective immunity in both adult and newborn pigs with only a single dose	Pre-clinical BALB/c mice Female pregnant pigs (*Susscrofa domesticus*) Hungarian large white pig	[128]
LVRNA001	RABV	RABV-G	Non-replicating mRNA vaccine encoding the RABV-G	Induced strong humoral and Th-1-derived cellular immune responses in mice Immunized mice and dogs were completely protected following an intracerebral challenge with a highly virulent RABV strain	Pre-clinical BALB/c mice Dogs	[129]
LVRNA001	RABV	RABV-G	Non-replicating mRNA vaccine encoding the RABV-G	Two LVRNA001 doses elicited strong immune responses in pre- and post-exposure scenarios and conferred complete protection in dogs No significant adverse effects in immunized cynomolgus macaques No toxicity observed in immunized Sprague-Dawley rats	Pre-clinical Cynomolgus macaques Dogs Sprague-Dawley rats	[130]
mRNA-LNP vaccine	RABV	RABV-G	Unmodified mRNA-LNP vaccine encoding the RABV-G	Elicited higher levels of neutralizing antibodies, memory B-cells, plasma cells, and T-cells in comparison to the licensed inactivated RABV vaccine, Rabipur Provided broader and durable immune responses	Pre-clinical Rhesus macaques	[131]
mRNA-based vaccine	RABV	RABV-G	mRNA-LNP vaccine encoding the RABV-G, encapsulated in a novel muscle-targeting LNP based on proprietary STAR-002 LNP formulation	Elicited high virus-neutralizing antibody and IgG titers in a dose-dependent manner A single dose was sufficient to confer 100% and 60% protection in pre-exposure and post-exposure challenges, respectively	Pre-clinical BALB/c mice	[132]
CV7201 (CureVac)	RABV	RABV-G	Lyophilized, thermostable mRNA vaccine encoding the RABV-G and formulated with a cationic protein, protamine	Highly immunogenic, but the generation of a sufficient immune response was highly dependent on the mode of administration Deemed to be safe and well-tolerated in immunized participants	Phase I NCT02241135 (Completed)	[133]
CV7202 (CureVac)	RABV	RABV-G	Similar to CV7201 but utilizes a novel mRNA-LNP formulation	Elicited RABV-specific neutralizing antibody responses even at low doses (1 µg or 2 µg) A 5 µg dose of CV7202 resulted in unacceptable reactogenicity in vaccinees	Phase I NCT03713086 (Completed)	[134]
ZIKV mRNA-LNP vaccine	ZIKV	Pre-membrane (prM) and envelope (E) structural proteins	Nucleoside-modified mRNA-LNP vaccines expressing the prM and E proteins	Elicited high neutralizing antibody titers (~1/100,000) in all three mouse models Provided protection in all three-immunized mice models against lethal ZIKV challenge	Pre-clinical AG129 mice BALB/c mice C57BL/6 mice	[137]
mRNA-1325 (Moderna) (1st generation ZIKV mRNA vaccine)	ZIKV	prM and E proteins from a Micronesia 2017 ZIKV strain	mRNA vaccine encoding the prM and E proteins	Well-tolerated among the immunized participants, but elicited poor ZIKV-specific neutralizing antibodies	Phase I NCT03014089 (Completed)	[138,139]
mRNA-1893 (Moderna) (2nd generation ZIKV mRNA vaccine)	ZIKV	prM and E proteins from a contemporary ZIKV strain (RIO-U1)	mRNA vaccine encoding the prM and E proteins	Two doses of mRNA-1893 were well-tolerated and elicited robust neutralizing antibodies in immunized participants	Phase I NCT04064905 (Completed) Phase II NCT04917861 (Completed)	[138,139]
DENV-2 mRNA-LNP vaccine	DENV	prM-E, 80% envelope protein (E80), NS1 of DENV-2 serotype	Three nucleotide-modified mRNA-LNP vaccines encoding prME-mRNA, E80-mRNA, or NS1-mRNA of DENV-2 serotype	Each vaccine candidate demonstrated robust immunogenicity by eliciting strong neutralizing antibodies, DENV-2 specific IgG, and antigen-specific T-cell responses The E80-mRNA vaccine alone or combined with the NS1-mRNA vaccine elicited high levels of neutralizing antibodies and conferred complete protection against DENV-2 challenge	Pre-clinical BALB/c mice	[142]
prM/E mRNA-LNP vaccine	DENV	prM and E proteins of DENV-1 serotype	Nucleotide-modified mRNA-LNP vaccine encoding the DENV-1 prM and E proteins	Elicited cellular and humoral immunity with neutralizing antibody titers that were sufficient for protection against DENV-1 Induced elicited serotype-specific antibody responses with minimal cross-reactivity to other DENV serotypes, reducing the risk of ADE	Pre-clinical AG129 mice C57BL/6 mice	[143]
DENV E-DIII + NS1 mRNA-LNP vaccine	DENV	Envelope E-DIII domain and NS1	DENV mRNA-LNP vaccine candidate containing DENV-a vaccine construct (DENV-1 E-DIII +DENV-2 NS1) and DENV-b vaccine construct (DENV-4 E-DIII + DENV-3 NS1)	Elicited high levels of neutralizing antibody titers against all four DENV serotypes with minimal ADE observed Induced elevated cytokine productions (TNF-α, IFN-γ), indicating Th-1 biased responses	Pre-clinical C57BL/6 mice	[144]
RVFV mRNA-LNP vaccines	RVFV	RVFV Gn and Gc glycoproteins	Six nucleoside-modified mRNA-LNP vaccine candidates encoding different regions of the RVFV Gn and Gc glycoproteins	mRNA-LNP vaccine candidate expressing the RVFV full-length Gn and Gc glycoproteins elicited robust humoral and cellular immune responses in mice, including conferring protection from lethal RVFV challenge Induced potent neutralizing antibodies, antigen-specific T-cell responses, and humoral memory B-cells in rhesus macaques	Pre-clinical BALB/c mice Rhesus macaques	[147]
RVFV mRNA-LNP VEEV genome-based vaccines	RVFV	RVFV Gn and Gc glycoproteins	Two mRNA-LNP VEEV genome-based vaccine candidates encoding the wild type RVFV Gn/Gc or modified (furin-T2A) Gn/Gc glycoproteins	Both vaccine candidates induced high levels of RVFV Gn-specific IgG antibodies in a dose-dependent manner Wild type RVFV vaccine candidate elicited pseudovirus-neutralizing activity whilst the modified vaccine candidate displayed reduced neutralization capacity	Pre-clinical BALB/c mice	[148]
POWV mRNA-LNP vaccine	POWV	POWV prM and E genes	Nucleoside-modified mRNA-LNP vaccine candidate encoding the POWV prM and E genes	Induced high titers of neutralizing antibody responses and protected mice from lethal challenges from several POWV strains Elicited cross-neutralizing antibodies against other tick-borne flaviviruses (TBFV), such as Langat virus Protected mice from Langat virus-induced disease	Pre-clinical C57BL/6 mice	[152]
CCHFV mRNA-LNP vaccines	CCHFV	CCHFV Gn/Gc glycoproteins or CCHFV nucleoprotein (N)	Two nucleoside-modified mRNA-LNP vaccine candidates expressing the CCHFV Gn/Gc glycoproteins or N	Induced robust humoral and cellular immune responses and provided complete protection against lethal CCHFV infection in A129 mice	Pre-clinical A129 IFNAR^-/-^ mice 129S2 mice	[158]
repGc/repNP RNA vaccine	CCHFV	CCHFV Gc (repGc) and NP (repNP)	Alphavirus-based self-replicating RNA vaccine candidate expressing the CCHFV repGc and repNP proteins	Elicited non-neutralizing NP antibodies and Gc- specific T-cell responses Immunized mice were protected against lethal CCHFV challenge for a year, with 100% survival up to 9 months and 80% survival within 1 year post-vaccination	Pre-clinical C57BL/J mice	[159]
repGc/repNP RNA vaccine	CCHFV	CCHFV repGc and repNP	Alphavirus-based self- replicating RNA vaccine candidate expressing the CCHFV repGc and repNP proteins	Two-dose vaccinated rhesus macaques elicited robust non-neutralizing humoral and cellular immune responses Provided significant protection against CCHFV challenge with the CCHFV strain Hoti	Pre-clinical Rhesus macaques (*Macca mulatta*)	[160]
CCHFV mRNA-LNP vaccines	CCHFV	CCHFV Gn and Gc glycoproteins	Three nucleoside-modified mRNA-LNP vaccine candidates encoding the Gn (vLMn), Gc (vLMc) or with Gn linked to Gc with an NSm linker (vLMs)	vLMc vaccine candidate elicited stronger B-cell and T-cell immune responses when compared to vLMn and vLMs candidates vLMc elicited higher Gc-specific IgG titers and IFN-γ T-cell responses	Pre-clinical BALB/c mice C57BL/6J	[161]
SFTSV mRNA-LNP vaccines	SFTSV	SFTSV soluble Gn head region (sGn-H) and sGn-H fused with 24-mer ferritin (FT) (sGn-H-FT)	Two mRNA-LNP vaccine candidates encoding the sGn-H and sGn-H-FT	Both vaccine candidates induced potent and durable neutralizing antibody responses lasting up to 12 weeks following a booster dose, with sGn-H-FT displaying slightly higher immunogenicity Immunization with either vaccine candidate completely protected mice from lethal SFTSV challenge	Pre-clinical A129 IFNAR^-/-^ mice BALB/c mice	[172]
SFTSV Gn mRNA-LNP vaccine	SFTSV	SFTSV Gn glycoprotein	mRNA-LNP vaccine candidate expressing the SFTSV Gn glycoprotein	Elicited robust neutralizing antibodies and Gn-specific T-cell responses, including increased frequency of Tfh cells Immunized mice were fully protected from lethal SFTSV challenge, with minimal weight loss, reduced liver and spleen damage observed	Pre-clinical C57BL/6 mice	[173]
Nucleoside- modified mRNA-LNP vaccine	SFTSV	SFTSV full-length Gn+Gc glycoproteins	Nucleoside-modified mRNA-LNP vaccine candidate encoding the SFTSV full-length Gn+Gc glycoproteins	Induced robust humoral response via generation of high titers of neutralizing antibodies and Th-1 biased responses in immunized mice Low or high doses of the vaccine provided complete protection in immunized mice from lethal SFTSV challenge and durable immunity for five months Conferred cross-protection against other *bandaviruses* like Heartland virus and Guertu virus	Pre-clinical BALB/c mice	[174]
LASV mRNA-LNP vaccines	LASV	LASV wild type or prefusion- stabilized glycoprotein complex (GPC)	Two nucleoside-modified mRNA-LNP vaccine candidates expressing the wild type or prefusion- stabilized GPC of LASV strain Josiah	Both vaccine candidates elicited robust binding antibody responses, specifically to the vaccine candidate containing the prefusion-stabilized GPC Neutralizing antibodies were interestingly detected in several of the guinea pigs Guinea pigs immunized with either vaccine candidate were protected from a lethal dose of the LASV strain Josiah without any severe disease manifestations or deaths, indicating that protection might be linked with Fc-mediated effects	Pre-clinical Hartley outbred guinea pigs	[180]
LASV mRNA-LNP vaccines	LASV	LASV glycoprotein precursor (LASgpc) or lymphocytic choriomeningitis virus nucleoprotein (LCMnp)	Two nucleoside-modified mRNA-LNP vaccine candidates expressing the LASgpc or LCMnp	Both vaccine candidates conferred protection from lethal lymphocytic choriomeningitis virus (LCMV), despite inducing negligible neutralizing antibodies Two doses of the vaccine candidates elicited robust humoral and cell-mediated immune responses, with the protective effect suggested to be associated with anti-LASV CD8^+^ T-cell responses	Pre-clinical C57BL/6N mice CBA/N mice	[181]

## Data Availability

Not applicable.
